# DNA methylation remodeling reveals epigenetic regulation of early embryogenesis in *Arabidopsis* hybrid

**DOI:** 10.1038/s42003-026-10245-5

**Published:** 2026-05-15

**Authors:** Wenjing Wang, Yingzhang Huang, Chunhui Hou, Dianjing Guo

**Affiliations:** 1 State Key Laboratory of Agrobiotechnology and School of Life Science, The Chinese University of Hong Kong, Hong Kong, China; 2 State Key Laboratory of Genetic Evolution & Animal Models, Kunming Institute of Zoology, Chinese Academy of Sciences, Kunming, China; 3 Yunnan Key Laboratory of Biodiversity Information, Kunming, China; 4 KIZ-CUHK Joint Laboratory of Bioresources and Molecular Research in Common Diseases, Kunming Institute of Zoology, Chinese Academy of Sciences, Kunming, China

**Keywords:** Plant embryogenesis, DNA methylation

## Abstract

Plant embryogenesis is a critical process characterized by extensive cellular differentiation and morphogenesis, regulated by complex epigenetic mechanisms. However, dynamic changes of DNA methylation during *Arabidopsis* early embryo development remain to be elucidated. In this study, *Arabidopsis* hybrid embryos from 2/4-cell to globular stage were subject to bisulfite sequencing and RNA-seq analysis. Our results revealed a local remodeling of DNA methylation during early embryogenesis, represented by a remarkable gain of CHH methylation and the progressive loss of CHG methylation. Genes with differential methylation were found to participate in cell division, morphogenesis, pattern specification and auxin signaling, indicating their potential role in embryo development. At globular stage, CHH hyper-methylation exhibited a notable association with TE repression. The divergences between allelic methylation primarily lied in CG context which increased with embryogenesis. CHH methylation variations between parental alleles underwent reprogramming. Our results implied a negative relevance between allele-specific expression and methylation at CHG sites in early embryos. This work provides new insights into DNA methylation remodeling and its association with transcriptional changes during *Arabidopsis* early embryogenesis, laying a foundation for future functional studies.

## Abstract (web summary)

Profiling of DNA methylation provides insights into its remodeling and potential roles in embryo development and transcriptional regulation during early embryogenesis in *Arabidopsis*.

## Introduction

Embryogenesis lays the foundation for understanding the development from a fertilized egg cell to a complex seedling. In *A. thaliana*, two sperm cells fertilize the egg cell and central cell, which then develop into the zygote and endosperm, respectively. After fertilization, asymmetrical division occurs to the elongated zygote, leading to the formation of an apical cell which generates the embryo, and a basal cell which develops into the suspensor. *Arabidopsis* embryo undergoes rapid cell divisions with limited expansion in embryo size until 8-cell stage, accompanied by the formation of upper and lower tiers. The radial axis is then established, and the protodermal cells are generated at 16-cell stage. The specification of vascular and ground tissues begins at the early-globular (32-cell) stage. After asymmetrical division of the hypophysis, the precursor of the quiescent centre (QC) is formed at the late-globular stage, contributing to the initiation of root apical meristem^1–3^. During maturation process, embryo undergoes rapid cell division and expansion, along with complex metabolism and morphogenesis^4^.

DNA methylation is one of the basic epigenetic mechanisms that participates in multiple biological processes to mediate the expression of genes and transposable elements (TEs). In plants, DNA methylation on cytosines can be detected in the contexts of CG, CHG and CHH, where H represents A, T, or C. Multiple pathways have been reported underlying the establishment and maintenance of DNA methylation in *A. thaliana*. DNA METHYLTRANSFERASE 1 (MET1) contributes to the maintenance of CG methylation in the daughter strand during DNA replication^5^. CHG methylation is maintained by CHROMOMETHYLASE 3 (CMT3) and CMT2 through a feedback loop between CHG and H3K9 methylation^6,7^. Maintenance of CHH methylation relies on CMT2 and DOMAINS REARRANGED METHYLTRANSFERASE 2 (DRM2) through RNA-directed DNA methylation (RdDM) pathway^8,9^. DECREASED DNA METHYLATION1 (DDM1) as a chromatin remodeler allows the access of DNA methyltransferases to the DNA, and facilitates CMT2-mediated CHH methylation^8^. RdDM pathway enables the DNA methylation in all three contexts of CG, CHG and CHH. Canonically, transcripts from RNA polymerase IV (Pol IV) are processed into double-stranded RNAs (dsRNAs) by RNA-dependent RNA polymerase 2 (RDR2). After the cleavage by DICER-LIKE3 (DCL3), 24-nucleotide small interfering RNAs (siRNAs) are loaded on ARGONAUTE4 (AGO4) and AGO6, and paired with transcripts from RNA polymerase V (Pol V). DRM2 is then recruited to catalyze cytosine methylation^9,10^. The failure of DNA methylation maintenance during DNA replication leads to a passive reduction of methylation levels. Moreover, the removal of DNA methylation can be actively catalyzed by four DNA demethylases in *Arabidopsis* including TRANSCRIPTIONAL ACTIVATOR DEMETER (DME), REPRESSOR OF SILENCING 1 (ROS1), DEMETER-LIKE 2 (DML2), and DML3^9^.

Efforts have been made to understand the zygotic transcriptome in *Arabidopsis*. Autran et al. reported a maternally dominant transcription in 2/4-cell embryos^11^, while parental genomes were found to contribute equally to the embryonic transcriptome initiating from 1/2-cell stage^12^. Moreover, a two-phase zygotic genome activation (ZGA) was demonstrated in *Arabidopsis* zygotes, indicating the equal parental contributions in the elongated zygote^13^. On the contrary, a reanalysis of early embryo transcriptomes in *Arabidopsis* hybrids revealed that hybridization may regulate the parental genome contributions to ZGA, exhibiting a direction-dependent asymmetry in crosses between Col and Ler accessions^14^.

Previous studies have illustrated the crucial roles of DNA methylation in plant development, e.g. the loss of CG methylation was found to impair normal seed development in *Arabidopsis*^15^. No embryo lethality occurred in *Arabidopsis* lacking non-CG methylation, shown as the normal embryonic morphogenesis and seed germination^16^, whereas transient patterning defects have been reported in non-CG methylation mutants^11,17^. The essential roles of DNA methylation in embryogenesis were demonstrated by the extreme developmental defects in DNA methylation-free *Arabidopsis*, in which CG and non-CG methylation jointly contributed to the regulation of meristem activity^18^. However, the overall small size of seeds, combined with the deeply embedded embryo within the endosperm and seed coat, make it challenging to isolate *Arabidopsis* early embryos without contamination. Knowledge has been limited in understanding the epigenetic regulation and allelic methylation divergences during early embryogenesis in *Arabidopsis*. Previous studies mainly focused on late embryogenesis (from globular embryos onwards), and revealed a gain of CHH methylation during seed development in *Arabidopsis* and soybean^16,19^. The progressive increase of CHH methylation in *Arabidopsis* embryos correlated with the accumulation of sRNAs during embryo development and an enhancement of RdDM activity during embryo maturation^20^. Embryonic CHH hyper-methylation was observed at both embryo-specific loci and shared loci that were less methylated in leaves^21^. Encouragingly, recent advancements in embryo isolation techniques allow us to focus on the early developmental stages^22,23^. Lately, study in rice revealed that DNA methylation underwent reconfiguration after fertilization. The remodeling of paternal allelic DNA methylation occurs to fit the maternal allele in rice zygotes^24^. Besides, maternal endosperm-derived sRNAs may play a role in TE silencing in early embryos, which is crucial for protecting genome stability^21^.

In this study, we utilized bisulfite sequencing (BS-seq) technology in *A. thaliana* hybrid embryos to investigate the dynamic changes of genome-wide DNA methylation during early embryogenesis. The integrated analysis with the embryonic transcriptome data revealed the impacts of DNA methylation remodeling on gene activity. Moreover, the allele-specific analysis provided new insights to the epigenetic regulation of allelic gene expression during *Arabidopsis* embryonic development.

## Results

### Gain of CHH methylation and loss of CHG methylation occurred concurrently during early embryogenesis

Cell elongation and division occur during early embryogenesis in *Arabidopsis*, along with distinguishable morphogenic changes. According to the time after pollination and the embryo morphology, we manually isolated ColxLer hybrid embryos at 2/4-cell, 8-cell, 16-cell, 32-cell, and globular stage as representative developmental stages. To characterize the genome-wide DNA methylomes during early embryogenesis, we performed bisulfite sequencing with two biological replicates, using low-input cells (Supplementary Table 1). To demonstrate the quality of our data sets, principal component analysis (PCA) was applied. Two biological replicates of each stage were clustered together, following the trajectory of development (Supplementary Fig. 1A). The calculation of Pearson correlation coefficient further confirmed the clustered biological replicates among time-series DNA methylation data (Supplementary Fig. 1B). In addition, DNA methylation levels exhibited strong correlation between biological replicates across all three contexts (*R* value ranging from 0.95 to 0.99, Supplementary Fig. 1C). These findings indicated the reliability of our data.

**Fig. 1:**
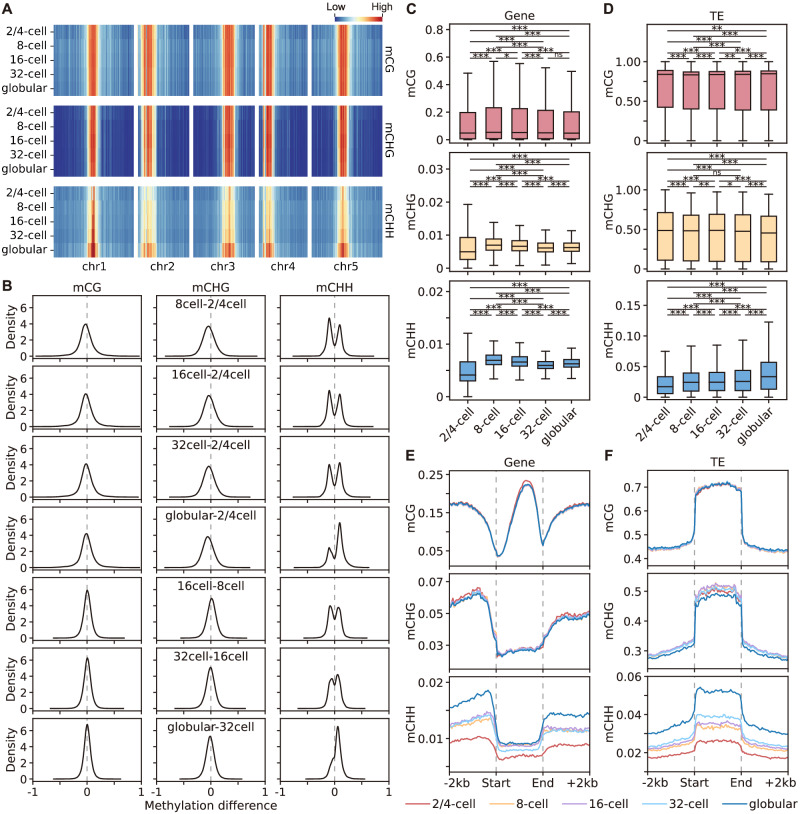
Profiling of genome-wide DNA methylation in *Arabidopsis* early embryos. **A** Estimation of DNA methylation levels along the genome in 100-kb bin size. **B** Density plots showing methylation differences with 100-bp bin size in early embryos relative to 2/4-cell stage, as well as pairwise comparisons between adjacent stages. Boxplots showing DNA methylation levels of protein-coding genes (**C**) and transposable element (TE) bodies (**D**). The box spanned from the first quartile (Q1) to the third quartile (Q3). The central line inside the box represented the median. The whiskers extended to the most extreme data points within 1.5 times the interquartile range (IQR) from the first and third quartiles. Significance was assessed by Mann–Whitney U test; thresholds: *p < 0.05, **p < 0.01, ***p < 0.001, ns meant not significant. Metaplots showing DNA methylation levels across protein-coding genes (**E**) and TEs (**F**).

Global profiling of DNA methylation in 100-kb bins revealed that the changes of DNA methylation were different for three contexts (Fig. 1A). Genome-wide CG and CHG methylation were largely remained during early embryogenesis, whereas CHH methylation was dramatically enhanced especially at globular stage (Fig. 1A). Furthermore, density plots revealed the methylation differences in 100-bp bins during embryogenesis. The decline in CG methylation mainly occurred in 8-cell versus 2/4-cell stage, with slight differences thereafter (Fig. 1B). CHG methylation was gradually weakened in developing embryos, except for a transient increase at the transition from 8-cell to 16-cell stage (Fig. 1B). Consistently, density plots showed a remarkable hyper-methylation for CHH context at globular stage (Fig. 1B).

Next, methylation changes around genes and TEs were investigated. A significant increase of DNA methylation for genes was detected right after 2/4-cell stage, followed by a progressive decline during early embryogenesis, with the exception of elevated CHH methylation levels in globular embryo (Fig. 1C). The profiling of DNA methylation across genes showed that an overall gain of CHH methylation occurred at 8-cell stage, whereas the enhancement at globular stage mainly targeted the flanking regions of genes (Fig. 1E). This was different from TEs, which exhibited a consistently strengthened CHH methylation during early embryogenesis (Fig. 1D, F).

**Fig. 2:**
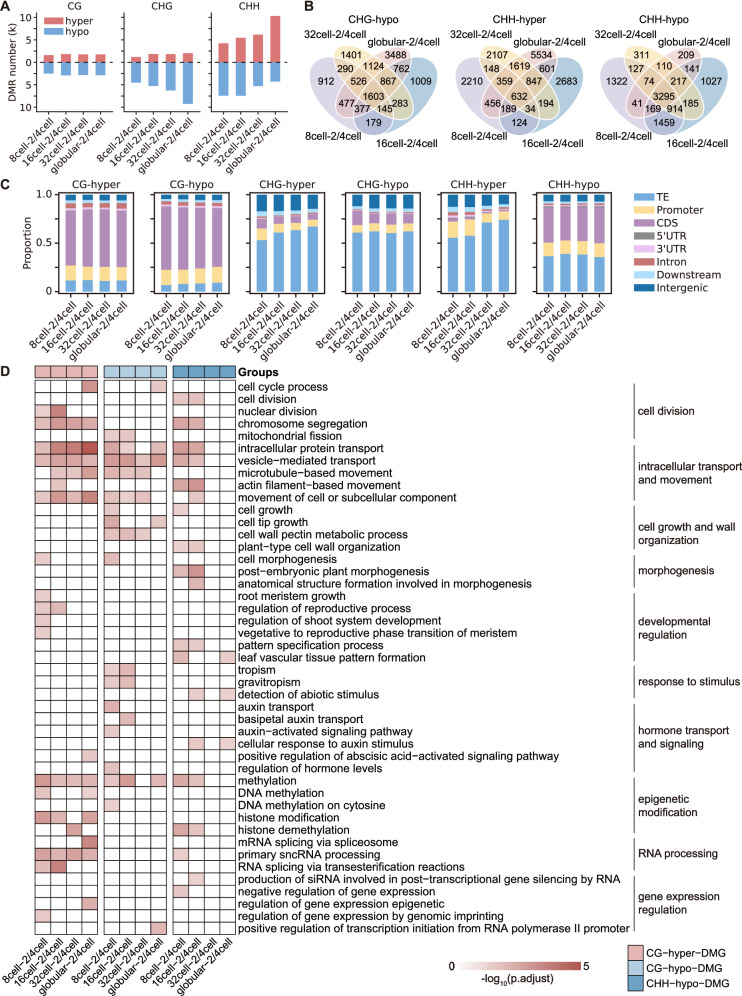
Local remodeling of DNA methylation during early embryogenesis. **A** Number of differentially methylated regions (DMRs) identified in early embryos versus 2/4-cell stage. **B** Venn diagram showing the overlaps of identified DMRs. **C** Annotation of DMRs distribution with genomic features. The promoter region was defined as 1000-bp upstream from the transcription start site (TSS). The downstream region was defined as 1000-bp downstream from the transcription end site (TES). **D** Gene Ontology (GO) enrichment analysis of genes with DMRs (DMGs) in biological processes (FDR < 0.05). The colorbar represented -log_10_(FDR).

To investigate the dynamics of DNA methylation in developing embryos, differentially methylated regions (DMRs) were identified using a sliding 100-bp window with 25-bp step size (Supplementary Data 1). In 8-cell versus 2/4-cell stage, more hypo-DMRs (2506 CG, 4546 CHG and 7423 CHH) than hyper-DMRs (1606 CG, 1172 CHG and 4230 CHH) were detected (Fig. 2A). Later in globular versus 2/4-cell stage, there were comparable number of CG DMRs (1761 hyper and 2858 hypo), whereas more CHG hyper-DMRs (2021) and much higher number of CHG hypo-DMRs (9250) were detected (Fig. 2A). CG hyper- and hypo-DMRs covered around 0.22–0.25 Mb and 0.34–0.4 Mb, which were highly conserved across pair-wise comparisons (Supplementary Fig. 2A, B), suggesting a moderate and stable remodeling of CG methylation during early embryogenesis. For CHG context, genomic coverage of hypo-DMRs increased from 0.64 Mb to 1.42 Mb (Supplementary Fig. 2A). 79.77% of CHG hypo-DMRs in 8-cell versus 2/4-cell stage can also be detected at later stages, whereas 37.81% of CHG hypo-DMRs at globular versus 2/4-cell stage were newly generated (Fig. 2B), suggesting a gradual occurrence of CHG hypo-methylation in developing embryos.

At globular versus 2/4-cell stage, much more CHH hyper- (10324) but less hypo-DMRs (4300) were detected (Fig. 2A). Correspondently, CHH hyper-DMRs occupied 0.61, 0.8 and 1.05 Mb in early embryos versus 2/4-cell stage, which strikingly increased to 2.23 Mb in globular versus 2/4-cell stage (Supplementary Fig. 2A). Notably, 53.23% of CHH hyper-DMRs were specially detected in 8-cell versus 2/4-cell stage, and similar proportion can be found in globular versus 2/4-cell stage, although the number was enlarged (Fig. 2B). These data suggested that variable CHH hyper-methylation occurred locally during early embryogenesis and a considerable enhancement occurred at globular stage. By contrast, 78.87% of CHH hypo-DMRs in 8-cell versus 2/4-cell stage were preserved at 16-cell versus 2/4-cell stage, and only 56.45% of them were consistently detected at later stages (Fig. 2B), revealing a gradual reduction of early established hypo-methylated regions in CHH context. Taken together, these results indicated that DNA methylation was locally remodeled during early embryogenesis.

To uncover the genomic elements that were relevant to the remodeled DNA methylation, we annotated the identified DMRs with TEs and multiple genic features. CG and CHG DMRs were mainly distributed in CDS regions and TEs respectively (Fig. 2C). Interestingly, CHH hyper-DMRs show preference on TEs, contrary to hypo-DMRs in both TEs and genic regions (Fig. 2C). The increased proportion of CHH hyper-DMRs in TEs suggested that the reconfiguration of CHH hyper-methylation in developing embryos may have impacts on TEs (Fig. 2C). For DMRs in close association with genic regions, we isolated genes uniquely overlapping with DMRs (DMGs), which show high consistency in developing embryos (Supplementary Fig. 3A, B). These DMGs were characterized by Gene Ontology (GO) enrichment analysis. The results suggested their participation in biological pathways related to cell division, intracellular protein transport, cell growth, epigenetic modification, and regulation of transcription, whereas the enrichment for morphogenesis, pattern formation, developmental regulation, and auxin signaling was observed in limited groups (Fig. 2D). Collectively, these data strongly indicated that local remodeling of DNA methylation was likely associated with molecular events in early embryonic development.

To characterize a comprehensive view of DNA methylation changes in developing *Arabidopsis* embryos, methylomes for early embryos at 2/4-cell, 8-cell and 16-cell stages were compared with publicly available methylomes for embryos at pre-globular^20^, early heart^20^, early torpedo^25^, bent cotyledon^20^, mid-torpedo to early mature^26^, and mature green^27^ stages, as well as seedling^27^ and leaf^20^. Genome-wide methylomes were evaluated in 100-kb bins, revealing the loss of CHG methylation during embryogenesis and the gain of CHH methylation peaking at mature green stage (Supplementary Fig. 4A). Although CHH methylation levels were relatively low in early embryos, a significant increase occurred after 2/4-cell stage. This was followed by a more substantial rise starting from pre-globular stage during embryogenesis, which then declined in seedlings and leaves (Supplementary Fig. 4B). Similar trend of CHH methylation dynamics was observed for TEs (Supplementary Fig. 4C, D). These results uncovered a progressive accumulation of CHH methylation during embryogenesis, which initiated shortly after 2/4-cell stage, preceding the pronounced surge observed at later stages.

### Influence of DNA methylation remodeling on embryonic transcriptome

To investigate the potential mechanisms underlying the remodeling of DNA methylation, embryos at the same five developmental stages were isolated for RNA-seq analysis, and three biological replicates were prepared (Supplementary Table 1). Considering the frequent occurrence of RNA contamination from maternal tissues, the tissue enrichment test^28^ was applied to demonstrate the quality of our embryonic transcriptomes. Significant enrichment of embryo proper-specific transcripts was observed across all 15 transcriptomes, while isolated embryos from 8-cell to 32-cell stage also exhibited enrichment of suspensor-specific transcripts (Supplementary Fig. 5). No contamination from endosperm or seed coat tissues was detected (Supplementary Fig. 5).

**Fig. 3:**
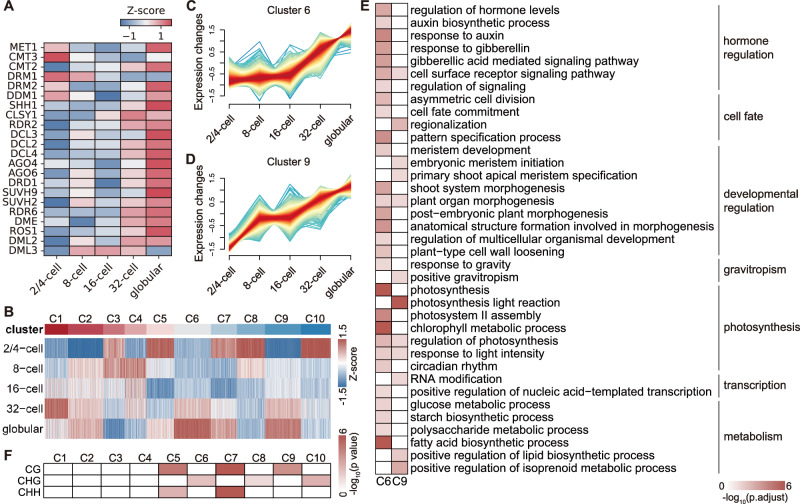
Dynamics of embryonic transcriptomes during early embryogenesis. **A** Expression levels of genes underlying the establishment, maintenance and removal of DNA methylation. Expression levels were calculated as log_2_(TPM + 1) with z-score normalization. **B** Expression levels of differentially expressed genes (DEGs) in 10 clusters using the R package Mfuzz, which were shown as log_2_(TPM + 1) with z-score normalization. Time-series expression changes of DEGs in cluster 6 (**C**) and cluster 9 (**D**). **E** GO enrichment analysis of DEGs in cluster 6 and cluster 9 (FDR < 0.05). The colorbar represented -log_10_(FDR). **F** Heatmap showing the significant overlap between DEGs and DMRs assessed by hypergeometric test. DMR-associated genes identified in developing embryos relative to 2/4-cell stage were pooled together, regardless of the direction of differential methylation. Next, expressed genes (TPM ≥ 1 in either stage) were isolated. Hypergeometric test was performed to estimate whether DEGs in each cluster were significantly associated with DMRs, compared to the expressed genes as the background. Only significant associations (*p* < 0.05) were shown.

We evaluated the transcripts of genes participating in the establishment, maintenance, and removal of DNA methylation. During early embryogenesis, the transcripts of *CMT3* peaked at 2/4-cell stage (Fig. 3A, Supplementary Fig. 6). Multiple DNA demethylation pathways were enhanced, represented by DNA demethylases *ROS1* (Fig. 3A, Supplementary Fig. 6). Notably, *CMT2* was almost undetectable at 2/4-cell stage but underwent transcriptional activation during embryogenesis (Fig. 3A, Supplementary Fig. 6). As a crucial participant of RdDM pathway, *DRM2* was steadily expressed during embryogenesis, especially at 2/4-cell and globular stage (Fig. 3A, Supplementary Fig. 6). Components of siRNAs biogenesis and de novo methylation (*CLSY1*, *RDR2*, *DCL2/4*, *AGO4/6*) were detected during embryogenesis, whose gene expression levels were elevated relatively at globular stage (Fig. 3A, Supplementary Fig. 6). These results suggested a decline in *CMT3* pathway activity during early embryogenesis, while the *CMT2*, RdDM and demethylation pathways were enhanced especially at globular stage.

To study the impact of differential methylation on gene expression, we estimated the transcripts of DMGs at globular versus 2/4-cell stage. Previously, a reverse relevance between CHG DMRs at gene body and their expression was reported in rice^29^. In soybean, the reduction in CHG methylation was associated with gene reactivation during somatic embryogenesis^30^. Such significant correlation between CHG hypo-methylation and up-regulation of DMG expression was also observed in our results (Supplementary Fig. 7A, B). Interestingly, the increased expression of DMGs widely occurred across three cytosine contexts at globular stage, under the condition of both hyper- and hypo-methylation (Supplementary Fig. 7A, B), suggesting a complicated relationship between DNA methylation remodeling and transcription. Considering the remarkable remodeling for CHH sites, up-regulated genes occupied by CHH DMRs were isolated and characterized by GO analysis. They were found enriched in several biological processes crucial for embryonic development, including cell morphogenesis, unidimensional cell growth, signaling, and auxin polar transport (Supplementary Fig. 7C).

To understand the dynamic changes of early embryonic transcriptome, 8543 differentially expressed genes (DEGs) were summarized into 10 clusters based on time-series expression using R package Mfuzz^31^ (Fig. 3B–D, Supplementary Fig. 8A, Supplementary Data 2). GO analysis was performed to annotate DEGs with distinct expression patterns. Auxin is crucial for the regulation of division orientation, the specification of cell types and pattern formation during embryogenesis^32^. Consistently, activated genes from 16-cell stage and thereafter in cluster 6 were enriched in auxin biosynthetic process, response to auxin, and pattern specification process (Fig. 3C, E). The asymmetrical division of the uppermost layer of suspensor cell occurs in early globular embryo, which is crucial for the initiation of root meristem^1^. Correspondently, asymmetric cell division showed enrichment in cluster 6 (Fig. 3C, E). Moreover, embryonic meristem initiation and development, shoot apical meristem specification and morphogenesis, together with photosynthesis-related processes, were enriched by activated genes in cluster 6 and 9 (Fig. 3C–E). In terms of repressed genes in cluster 8, they mainly contributed to the maintenance of DNA methylation and the regulation of heterochromatin organization (Supplementary Fig. 8A, B).

Next, we analyzed whether genes in distinct clusters were relevant to differential methylation during early embryogenesis. Both activated genes (in cluster 6 and 9) and repressed genes (in cluster 8 and 10) were found strongly associated with DMRs that occurred during embryogenesis (Fig. 3F). Additionally, DEGs in cluster 5 and 7 exhibited fluctuated expression, which were significantly overlapped with CG and CHH DMRs (Fig. 3F). Taken together, these results suggested a correlation between DNA methylation remodeling and transcriptional regulation during early embryo development.

### Relevance between TE methylation state and expression

**Fig. 4:**
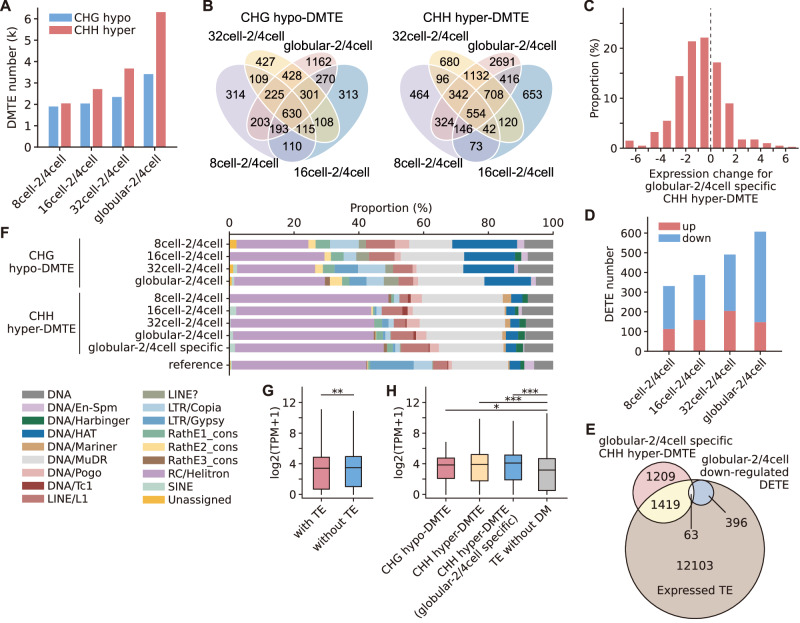
Influence of DNA methylation remodeling on TE expression in early embryos. **A** Number of TEs with DMRs identified in early embryos versus 2/4-cell stage. **B** Venn diagram showing the overlaps of identified DMR-associated TEs (DMTEs). **C** Proportion of CHH hyper-DMTEs that were newly identified in globular versus 2/4-cell stage, whose expression changes were distributed in distinct intervals, according to the log_2_FoldChange. **D** Number of identified differentially expressed TEs. **E** Venn diagram showing the overlaps between the newly identified CHH hyper-DMTEs and the down-regulated TEs in globular versus 2/4-cell stage. **F** Annotation of DMTEs that located within gene promoter regions. Genome-wide TEs were annotated as reference. **G** Boxplot showing gene expression differences at globular stage based on the presence of proximal TE in promoter regions. **H** Boxplot showing gene expression differences at globular stage based on epigenetic states of promoter-proximal TEs. Significance was assessed by Mann–Whitney U test; thresholds: *p < 0.05, **p < 0.01, ***p < 0.001.

The fact that DNA methylation is also crucial for TE silencing inspired our interests to investigate the effects of differential methylation on TE expression. Given that embryos at globular stage, a crucial developmental stage of embryogenesis during which tissue specification is initiated^1^, exhibited a notable CHH hyper-methylation within TEs compared to those at 2/4-cell stage, an early stage prior to extensive cell divisions (Fig. 1F), we focused on the variations between 2/4-cell and globular stage. Accordingly, DMR-associated TEs (DMTEs) were isolated, most of which were in CHG and CHH contexts (Fig. 4A). At globular versus 2/4-cell stage, remarkable increases occurred in the number of TEs with CHG hypo- and particularly CHH hyper-DMRs (Fig. 4A), concordant with the higher ratio of CHH hyper-DMRs annotated within TEs during embryogenesis (Fig. 2C). Relative to the earlier identified DMTEs, large proportion of CHG hypo- (1162 out of 3412) and CHH hyper-DMTEs (2691 out of 6313) were newly formed at globular versus 2/4-cell stage (Fig. 4B).

Next, we isolated DMTEs that were specifically identified at globular versus 2/4-cell stage. Excluding unexpressed TEs, the remaining DMTEs were largely down-regulated at globular stage (Fig. 4C). Moreover, 148 and 459 differentially expressed TEs (DETEs) were identified at globular versus 2/4-cell stage, along with a distinctive rise in the amount of down-regulated TEs (Fig. 4D). Interestingly, those TEs newly affected by CHH hyper-methylation at globular versus 2/4-cell stage were significantly overlapped with transcriptionally repressed TEs (*p* = 0.01896, hypergeometric test, Fig. 4E), indicating that the strengthened CHH methylation in globular embryo was relevant to the repression of TE expression.

TE insertions upstream of the gene are known to have potential impacts on the expression of nearby genes, by disruption of regulatory sequences or providing novel regulatory sites^33^. Besides, chromatin modifications of TEs may spread to the promoter regions and mediate nearby gene expression^33^. To investigate whether the changes in TE methylation were linked to transcriptional regulation of nearby genes, we isolated DMTEs that were located within gene promoters. After annotation, two groups of DMTEs displayed distinct proportional composition of TE classes, with particularly pronounced divergence in DNA/HAT, DNA/MuDR, LTR/Copia, LTR/Gypsy, and RC/Helitron (Fig. 4F). It’s well known that genes with the presence of proximal TEs always exhibit lower expression levels compared to those without TEs^34^. Consistently, our results confirmed this pattern (Fig. 4G). Moreover, genes show significantly higher expression levels at globular stage if promoter-proximal TEs displayed differential methylation (Fig. 4H). These results partially supported the influence of TE methylation remodeling on nearby genes.

### Increased divergences between allelic methylation during early embryogenesis

**Fig. 5:**
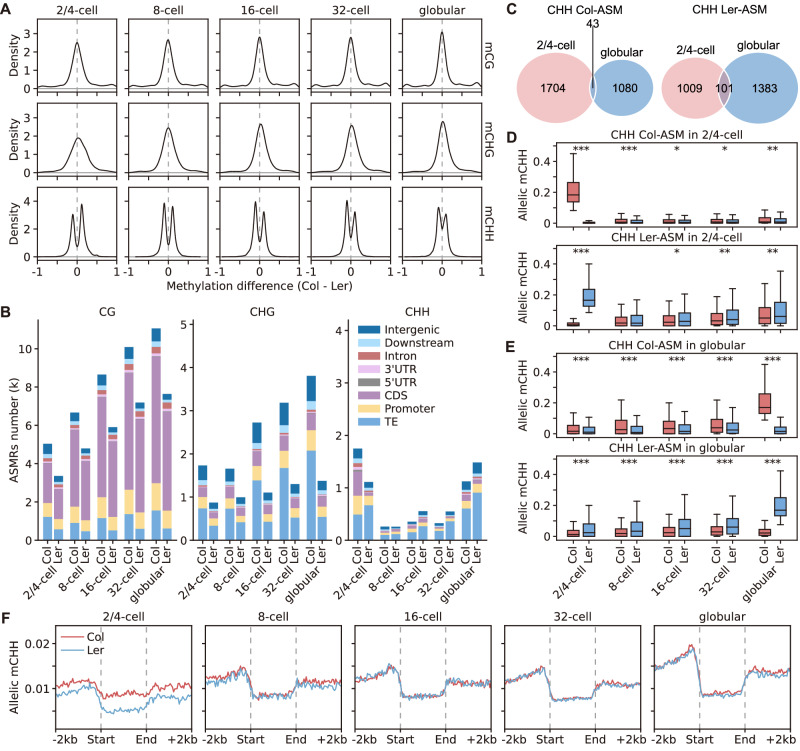
Dynamic variations between allelic methylation during early embryogenesis. **A** Density plots of methylation differences between two alleles in early embryos with 100-bp bin size. **B** Number of regions with allele-specific methylation (ASMRs) whose distribution was annotated with multiple genomic features. **C** Venn diagram showing the overlaps of ASMRs identified in CHH context. Boxplots tracking the changes of allelic CHH methylation levels, anchored by CHH ASMRs identified in Col (upper panel) or Ler (lower panel) from the embryos at 2/4-cell (**D**) and globular (**E**) stage. Allelic methylation levels for target regions were estimated across five stages. CHH methylation levels of Col and Ler allele were shown in red and blue. Significance was assessed by Mann–Whitney U test; thresholds: *p < 0.05, **p < 0.01, ***p < 0.001, ns meant not significant which was not shown. **F** Metaplots showing allelic CHH methylation levels across genes.

To study the dynamic changes of methylation variations between parental alleles during embryogenesis, the parental origin of reads was assigned using SNP sites between Col and Ler, and the allelic methylation levels were estimated. Density plots for allelic methylation differences in 100-bp bins indicated that CHG methylation levels were elevated in Col allele compared to those in Ler allele (Fig. 5A). Notably, the identification of regions with allele-specific methylation (ASMRs) in CG and CHG context showed higher prevalence in Col compared to Ler allele (Fig. 5B, Supplementary Data 3). CG ASMRs were largely distributed in genic regions, constituting the majority of allelic methylation variations in early embryos (Fig. 5B, Supplementary Fig. 9A). CG ASMRs identified at 2/4-cell stage were highly conserved, whereas hundreds of novel CG ASMRs emerged in developing embryos (Fig. 5B, Supplementary Fig. 9B). Similar trends were found in CHG context, which occupied a smaller proportion of ASMRs and primarily targeted TEs (Fig. 5B, Supplementary Fig. 9). The progressive increase in ASMR numbers on both alleles revealed the intensified variations between allelic methylation in CG and CHG context during early embryogenesis.

It should be noted that, allelic CHH methylation exhibited a reversal in the relative heights of peaks during embryogenesis (Fig. 5A), suggesting a transition in methylation preference from Col allele to Ler allele. Col allele contributed more CHH ASMRs than Ler allele at 2/4-cell stage, and the CHH ASMRs subsequently experienced almost complete temporal erasure at 8-cell stage, followed by gradual re-emergence until remarkable re-establishment at globular stage (Fig. 5B, Supplementary Data 3). The re-emerged CHH ASMRs were more prevalent in Ler allele, and exhibited large differences from the initial ASMRs at 2/4-cell stage (Fig. 5B, C, Supplementary Fig. 9).

To study the dynamics of allelic methylation underlying the loss and re-establishment of ASMRs during embryogenesis, we specifically investigated the CHH ASMRs identified at 2/4-cell and globular stage. Regarding the initial ASMRs identified in Col allele, the higher allelic CHH methylation of Col allele was almost erased right after 2/4-cell stage (Fig. 5D). For ASMRs detected in Ler allele, allelic variations of CHH methylation were reduced by the progressively methylated Col allele and the concordantly changed Ler allele (Fig. 5D). Apart from the inconsistent dynamic pattern, initial CHH ASMRs in the two alleles also exhibited distinct distributions within genomic features, represented by the major preference within TEs for ASMRs in Ler than Col allele (Fig. 5B). In addition, in line with the distribution bias of initial ASMRs for genic regions, metaplots revealed higher allelic CHH methylation across genes in Col than Ler allele at 2/4-cell stage (Fig. 5F). Oppositely, considering the latest ASMRs identified in the two alleles, allelic CHH methylation shared similar trends of emergence during embryogenesis, accompanied with comparable genomic distribution (Fig. 5B, E). Taken together, these data implied that variations between allelic CHH methylation underwent a special reprogramming in early embryos.

### Emerged relevance between allelic gene expression and methylation

**Fig. 6:**
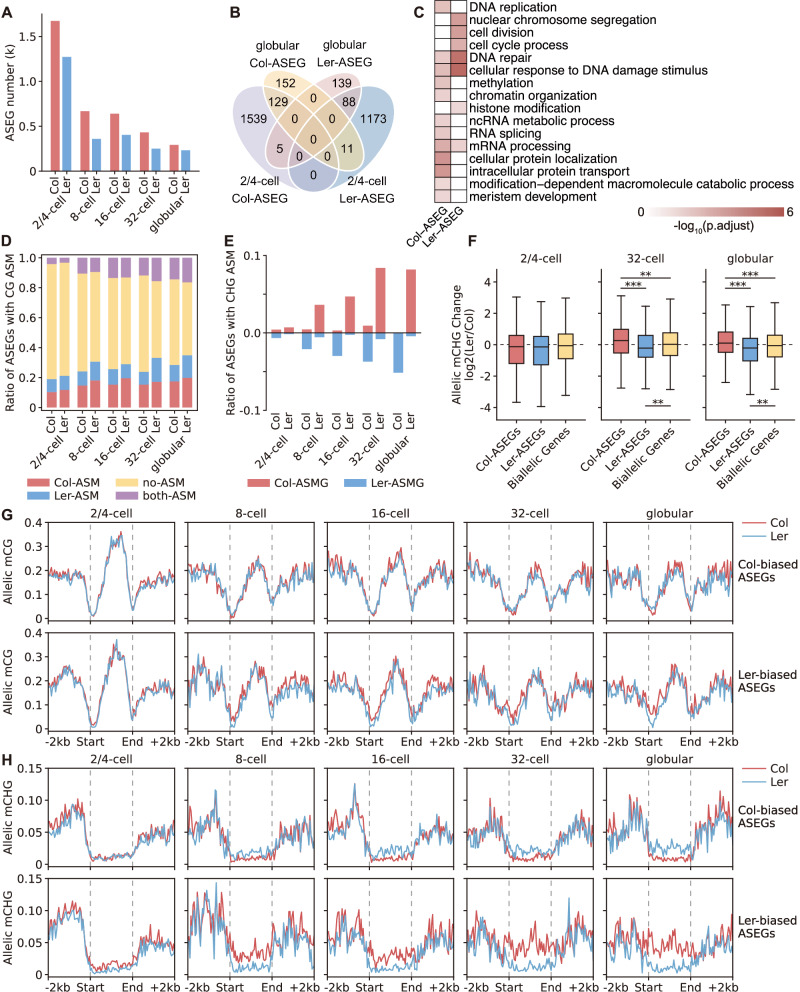
Association between allele-specific methylation and monoallelic expression. **A** Number of genes with allele-specific expression (ASEGs) identified in each allele. **B** Venn diagram showing the overlaps of ASEGs identified in 2/4-cell and globular stage. **C** GO enrichment analysis of ASEGs specifically identified in 2/4-cell stage (FDR < 0.05). The colorbar represented -log_10_(FDR). **D** For ASEGs identified in early embryos, the proportion of ASM-associated ASEGs was summarized in CG context. **E** For ASEGs identified in early embryos, the proportion of ASEGs containing CHG ASMs was calculated. Genes with ASM in Col and Ler allele were represented by red and blue. **F** Boxplots showing allelic CHG methylation differences for ASEGs and biallelic expressed genes identified in 2/4-cell, 32-cell, and globular stage. Significance was assessed by Mann–Whitney U test; thresholds: *p < 0.05, **p < 0.01, ***p < 0.001, ns meant not significant which was not shown. Metaplots showing allelic CG (**G**) and CHG (**H**) methylation levels across ASEGs identified in early embryos, respectively.

To study the dynamics of allele-specific expression, RNA-seq reads were assigned to parental origins based on the covered SNP sites. The results suggested a similar parental contribution to embryonic transcriptomes as early as 2/4-cell stage (Supplementary Fig. 10). In total, 1673 genes with allele-specific expression (ASEGs) in Col and 1272 in Ler allele were identified at 2/4-cell stage (Fig. 6A). However, only 292 ASEGs in Col and 232 in Ler allele were detected at globular stage (Supplementary Data 4). Among them, 44.18% (129 out of 292) and 37.93% (88 out of 232) exhibited a consistent bias of ASE (Fig. 6A, B). GO analysis suggested that ASEGs specifically identified at 2/4-cell stage were enriched in cell division, histone modification and meristem development (Fig. 6C). Interestingly, only 11 out of 292 (Col) and 5 out of 232 (Ler) ASEGs underwent identity switch compared to ASEGs at 2/4-cell stage (Fig. 6B). Among the genes with allelic expression preference switching from Col to Ler allele during embryogenesis, *PMEI3* encodes a pectin methylesterase inhibitor involved in the regulation of shoot apical meristem (SAM) development^35^.

To investigate the relationship between allele-specific expression and methylation, the ratio of ASEGs associated with ASMs were calculated. During early embryogenesis, ASEGs in Col and Ler allele displayed comparable allelic preference on CG ASMs, along with increasing proportion of genes containing ASM in both alleles (Fig. 6D). Among those ASEGs containing CG ASM at 2/4-cell stage, several SAM developmental regulatory genes (*ROCK1*, *LUG*, *ATBTAF1*, *LUH*) were identified. Next, we profiled the allelic CG methylation across ASEGs in Col and Ler allele, using genes with biallelic expression as comparison. At globular stage, ASEGs in Ler allele exhibited higher allelic CG methylation at gene body in Col compared with those in Ler allele, which was poorly detected in early embryos (Fig. 6G, Supplementary Fig. 11A). This finding suggested an ambiguous relevance between allelic expression and methylation in CG context.

Interestingly, ASEGs in Col allele were always coupled with CHG ASM in Ler allele, and the same applied in reverse for ASEGs in Ler allele and CHG ASM in Col allele (Fig. 6E). During early embryogenesis, the proportion of ASEGs containing CHG ASM grew constantly (Fig. 6E), which was in agreement with the profiling results of allelic CHG methylation across ASEGs. At 2/4-cell stage, similar performance for ASEGs in two alleles was uncovered (Fig. 6H). Thereafter, the allele with relatively higher expression always corresponded to weaker CHG methylation (Fig. 6H). In contrast, allelic CHG methylation was constantly comparable for biallelic genes in developing embryos (Supplementary Fig. 11B). The significance of allelic variations in CHG methylation was confirmed at globular stage, but not at 2/4-cell stage (Fig. 6F). These results indicated that allelic CHG methylation may exert negative impacts on allele-specific expression, which were progressively established in developing embryos after 2/4-cell stage.

## Discussion

Embryogenesis is a crucial developmental process accompanied by complex differentiation, pattern specification and morphogenesis in plants. Previous epigenetic studies provided partial knowledge of DNA methylation reconfiguration during late embryogenesis and germination. This work aims to investigate the dynamics of DNA methylation and its potential role in epigenetic regulation during *Arabidopsis* early embryogenesis. Our results indicated that local reconfiguration of DNA methylation was distinct across three sequence contexts (Fig. 1B). One remarkable remodeling was the strengthened CHH methylation especially at globular stage with preference on the TE regions, combined with the gradual disappearance of early established hypo-methylated CHH sites during embryogenesis (Fig. 2). Consistently, previous studies on seed development have observed the gain of CHH methylation especially around the TEs in *Arabidopsis*^19,27^, soybean^16^, and chickpea^36^. Such common phenomenon observed across plants species likely indicates an important evolutionary conservation. In *Arabidopsis*, both CMT2 and RdDM pathways are thought to remain active during seed development that contribute to TE hyper-methylation, whereas DNA demethylation occurs passively during germination^19^. Our results revealed enhancements in the CMT2 and heterochromatin pathways at globular stage, as well as an activated RdDM pathway during early embryogenesis (Fig. 3A, Supplementary Fig. 6). It’s likely that the early initiated reinforcement of CHH methylation from 2/4-cell stage is actively programmed by CMT2 and RdDM pathways.

Unlike the dynamic pattern of CHH methylation, a localized, moderate, and stable remodeling of CG methylation after 2/4-cell stage that mainly targeted genic regions was identified (Fig. 2). Previous study in *Arabidopsis* and soybean reported limited global changes on CHG methylation during embryo development from globular to mature stage^16^. In our study, local scanning for DMR revealed a progressively occurred localized CHG hypo-methylation during early embryogenesis. Such dynamic pattern might be regulated by multiple factors collectively. Given that CHG methylation is primarily maintained by CMT3 and CMT2, we observed a decline in the initially high expression of *CMT3* after 2/4-cell stage, in contrast to *CMT2* which was transcriptional activated during early embryogenesis (Fig. 3A, Supplementary Fig. 6). These findings suggested a weakened CMT3-mediated maintenance of CHG methylation in early embryos, which raised the possibility of passive dilution of CHG methylation, whereas targets of CMT2 pathway might be less affected. Moreover, enzymes responsible for DNA demethylation were up-regulated after 2/4-cell stage, providing an alternative hypothesis regarding the actively programmed local reduction of CHG methylation during early embryogenesis. Future studies using mutants of DNA methyltransferases and demethylases are needed to explore the effective mechanisms underlying the changes of CHG methylation in early embryos.

DNA methylation, together with various histone modifications, mediates chromatin accessibility and regulates gene activity during development. Previous evidence in chickpea suggested the contribution of differentially methylated genes to seed development^36^. Consistently, our results indicated that genes with differential methylation were likely responsible for embryogenesis-related processes (Fig. 2D). This is supported by previous finding that during *Arabidopsis* embryogenesis, mutation of *MET1* led to abnormal cell division and improper establishment of auxin gradients since early stage^15^. Unlike in mammals where DNA methylation at promoter regions is solely related to gene repression, promoter methylation in plants seems to participate in both activation and inhibition of multiple genes, such as during fruit ripening in tomato and orange^9^. Relevance between gene body CHG differential methylation and their expression has been proposed in rice and soybean^29,30^. However, in this work the upregulation of DMGs at globular stage was detected not only for those with hypo-methylation of CHG, but also for those with either hyper- or hypo-methylation of CG and CHH (Supplementary Fig. 7A, B). Although the epigenetic regulation on embryonic transcription was complex, up-regulated genes with differential methylation in CHH context were enriched in cell morphogenesis, unidimensional cell growth, and auxin polar transport (Supplementary Fig. 7C), indicating their potential role during early embryogenesis. In addition, DEGs with distinct expression pattern and functions during embryogenesis were significantly associated with DNA methylation remodeling (Fig. 3F).

Another critical role of DNA methylation is TE silencing to protect genome stability^9^. A notable relevance between the enhancement of CHH hyper-methylation and TE repression at globular stage was observed (Fig. 4). This is consistent with previous finding that loss of non-CG methylation was implied for TE de-repression^16^. Genes with promoter-proximal TEs tended to have lower expression levels (Fig. 4G), which might be due to the interrupted binding of transcription factors to the regulatory sequences. Nevertheless, TE insertions can also introduce novel promoters for nearby genes. Upon silencing by chromatin modifications, TE insertions within the promoter switched from blocking gene expression to instead producing transcripts from an outward-reading promoter provided by inserted TEs, which suppressed the mutant phenotype in maize. Moreover, expression of TEs was regulated by complex mechanisms during stress responses and development, while the regulatory sequences provided by TEs may act as enhancers or repressors and influence the transcription of nearby genes, with varying performances across TE families^33,37^. Apart from DNA methylation, recent studies revealed that H3K27me3 at TEs also contributed to transcriptional repression of TEs in flowering plants, representing an alternative TE silencing pattern^38^. Additionally, changes in chromatin states of TEs would interferer nearby gene expression^33^. The accessibility of chromatin around genes may also be affected by proximal TEs. In our data, the occurrence of methylation remodeling in promoter-proximal TEs was found to influence the expression of nearby genes (Fig. 4H), raising the possibility that the changes of chromatin modifications in TEs may exert local regulatory functions on nearby gene expression during early embryogenesis, whereas the exact mechanisms underlying such interplay remained complicated.

Epigenomes from parental origins revealed the dynamics of allelic methylation after fertilization (Fig. 5B). The CG context constituted the majority of allelic methylation variations from 2/4-cell stage, with an increasing trend during early embryogenesis. In a slightly different manner, variation in allelic CHG methylation increased by the emerged ASMRs mainly in maternal allele. Interestingly, differences between allelic CHH methylation underwent a special reprogramming during early embryogenesis. Previous study in rice illustrated that methylation remodeling reset the paternal epigenome to maternal state in zygote and 2-cell embryo, whereas the paternal ASM was re-established in globular embryo^24^. The distinctive remodeling especially in CHH context raises further questions about the inheritance of parental DNA methylation. Currently, isolating intact embryos at very early stages after fertilization remains technically challenging, due to their extremely small size, limited yield of early embryos released from siliques, and the risk of contamination from surrounding tissues. Optimization of low-bias library preparation for low-input cells would be beneficial for advancing such studies.

The utilization of hybrid embryos facilitated the investigation of allele-specific epigenetic differences, but also introduced genetic variations between parental genomes. Such variations collectively contribute to the transcriptional changes in hybrids compared to the parents^39^. In this study, we found a trend that the negative association between ASE and allelic CHG methylation became increasingly significant in early developing embryos (Fig. 6H). Previous studies have also reported similar findings, revealing the repressive impact on allelic expression by ASM at CHG sites in *Arabidopsis lyrata* and hybrid rice^29,40^. In *Arabidopsis* embryos, transcription of paternal alleles was regulated by the maternal epigenetic pathways, including non-CG methylation and the H3K9me2 histone methylation^11^. The H3K27me3 histone mark was also shown to regulate monoallelic expression in the embryo^41^. Therefore, the intensified allelic variations in CHG methylation may partially contribute to epigenetic regulation of monoallelic expression during early embryogenesis. Future work using reciprocal crossing may facilitate a better understanding of parental genome contributions to embryo development. Apart from allelic epigenetic variations, further studies are needed to demonstrate the potential impacts of genome heterozygosity on DNA methylation remodeling and gene activity in early embryos.

## Methods

### Plant materials and embryo isolation

*Arabidopsis thaliana* Col-0 and Ler were used for hybridization and embryo isolation to perform BS-seq and RNA-seq. Plants were grown at 22 °C with a 16/8 h light/dark cycle in a Panasonic growth chamber (MLR-325).

The isolation of *Arabidopsis* early embryos was performed based on previous methods^22,23,42^. Before hybridization, flowers of Col-0 were removed to avoid contamination. Then, flowers of Col-0 before opening were emasculated and hand pollinated with flowers from Ler after 24 h. Several hours after pollination, seeds at corresponding developmental stages were collected from siliques under a stereoscopic microscope (Olympus SZX16) and transferred into 50 μl washing solution (80 mM D-Mannitol (Sigma-Aldrich, M4125), 10% glycerol (Sangon Biotech, A501745-0500), 0.058% MES (Sigma-Aldrich, M3671) (pH 5.8)). After collecting multiple tubes of seeds, embryos were released by gently crushing the seeds with a plastic pestle. The droplets containing embryos were transferred onto the slides and screened under the Carl Zeiss PALM Inverted Microscope, and the embryos were collected and transferred to 100 μl washing solution using a homemade glass pipette. The collected embryos were washed twice with 100 μl washing solution until the background became clean, to avoid the contamination from maternal tissues. The collected embryos were cross-linked by 1% formaldehyde (Sigma-Aldrich, 252549) for 10 min, and then treated with 0.125 M Glycine (Sigma-Aldrich, G8898) for 5 minutes to stop reaction. After centrifuge under 1000 × *g* for 3 min, the cross-linked embryos were washed twice with 100 μl washing solution. Through the screening under the Inverted Microscope, the embryos at the same developmental stage were collected and stored at −80 °C freezer for BS-seq library construction. For RNA-seq, seeds were transferred to 50 μl enzyme solution (0.1% Cellulase R-10 and 0.08% Macrozyme R-10 (Yakult Pharmaceutical Ind. Co. Ltd., Japan) dissolved in washing solution) and treated for 15 min. Then, seeds were washed twice with washing solution, and resuspended for crushing with a plastic pestle. Lastly, the embryos were collected and stored without cross-linking.

### Bisulfite sequencing

Starting from the collected embryos with cross-linking, 32 μl lysis buffer (20 mM Tris-HCl (pH 7.4), 0.1% Triton X-100, 10 mM NaCl, 2 mM CaCl2) was added followed by 10 min incubation on ice. 8 μl diluted MNase (0.33 U/ml, Sigma-Aldrich, cat. No. N3755-200UN) was added and then incubated for 5 min at 37 °C. To stop the reaction, 80 μl stop buffer (10 mM Tris-HCl (pH 7.4), 150 mM NaCl, 10 mM EDTA, 0.15% SDS, 0.1% Triton X-100) and 2 μl protease K (TRANSGEN, cat. No. GE201-01) were added. After that, the samples were treated by 6 h incubation at 65 °C for the purpose of reverse crosslinking. DNA was extracted and purified using the phenol-chloroform extraction and ethanol precipitation. BS-seq libraries were prepared using the NEBNext Ultra II DNA Library Prep with Sample Purification Beads (NEB, E7103) and the NEBNext Multiplex Oligos for Illumina (NEB, E7535). Bisulfite conversion was performed using the EZ DNA Methylation-Gold Kit (ZYMO Research, D5005) following the manufacturer’s instructions. NEBNext Q5U Master Mix (NEB, M0597) was used to amplify the bisulfite-converted libraries. Finally, BS-seq libraries were sequenced using the Illumina platform with PE150.

### BS-seq data processing

Reads were filtered and trimmed using trim_galore v0.6.7 with default parameters. N-masked reference genome was generated based on the SNP information between Col and Ler^43^. Reads were then aligned to the N-masked reference genome and deduplicated using Bismark v0.23.1^44^. Finally, methylation levels for three cytosine contexts were extracted for further analysis. Only cytosines covered by at least 4 reads were considered. Metaplots to profile methylation levels across the interested regions were generated using deeptools^45^. For DMR screening, a sliding window with 100-bp bin size and 25-bp step size in methylKit^46^ was applied. Windows containing at least 4 cytosine sites were further considered. Fisher’s exact test was applied to select windows with an FDR < 0.05, along with an absolute methylation difference over 0.4, 0.2, 0.1 for CG, CHG, CHH, respectively. Windows within 100-bp were merged. DMGs were identified as the genes overlapped with either hyper- or hypo-DMRs, excluding those genes with conflicting overlap. Gene Ontology analysis was applied using the R package clusterProfiler v4.4.4^47^ using the parameter FDR < 0.05.

Allelic origins of the aligned reads were assigned using SNPsplit^48^, followed by the extraction of allelic methylation states. Only cytosines covered by at least 4 allelic reads were considered. The identification of allele-specific methylated regions was modified based on previous studies^49,50^. The significance of allelic methylation bias was evaluated by Fisher’s exact test and an FDR < 0.05 was accepted. An absolute difference over 0.4, 0.2, 0.1 for CG, CHG, CHH between allelic methylation was needed, respectively.

Publicly available methylomes for embryos at pre-globular^20^, early heart^20^, early torpedo^25^, bent cotyledon^20^, mid-torpedo to early mature^26^, and mature green^27^ stages, as well as seedling^27^ and leaf^20^, were downloaded and processed using a similar pipeline with one modification: reads were aligned to the reference genome without N-masking.

### RNA-seq library construction

mRNA was extracted from the collected embryos using the Dynabeads mRNA DIRECT Micro Kit (Life Technologies) following the manufacturer’s instructions. After that, the SMARTer Ultra Low RNA Kit for Illumina Sequencing (Clontech, USA) was used for cDNA synthesis and amplification by LD-PCR. Purification of amplified cDNA was performed with VAHTS DNA Clean Beads (Vazyme, N411-01, China), followed by sonication. The fragmented cDNA was used for RNA-seq library construction with the VAHTS Universal DNA Library Prep Kit for Illumina V3 (Vazyme, N607-01, China). Finally, RNA-seq libraries were sequenced using the Illumina platform with PE150.

### RNA-seq data processing

Low-quality reads and adapters were trimmed using cutadapt v3.7^51^, and the clean data was aligned to the N-masked reference genome using hisat2^52^. Only unique mapped reads were further considered. FeatureCounts^53^ was applied to count the aligned reads for genomic features including genes and TEs. TEs overlapped with genes were excluded to clarify the assignments of reads. Gene expression levels in each sample were quantified as transcripts per million (TPM), a normalization method that accounts for both gene length and sequencing depth. Differential expression of genes and TEs was identified using DESeq2^54^ with the parameters |log_2_FoldChange| > 1 and FDR < 0.05.

To evaluate the allele-specific expression, SNPsplit was applied to assign the parental origin of the aligned reads, and then allelic reads for genes were counted using FeatureCounts. According to the previous studies^55–57^, the allelic read counts were normalized using the size factor obtained from the total library size. Genes with at least 10 reads from either maternal or paternal allele in all replicates were further considered. Fisher’s exact test was applied to estimate the significance of allelic expression bias relative to the expectation. Genes with an FDR < 0.05 and at least 65% normalized reads from maternal or paternal allele were identified as monoallelic expression. In comparison, genes with an FDR > 0.05 and the allelic ratio ranging from 35% to 65% were identified as biallelic expression.

### Statistics and reproducibility

Mann–Whitney U test was applied to Figs. 1C, D, 4G, H, 5D, E and 6F, Supplementary Figs. 4B, C, 7B. Levels of significance were indicated as follows: *p < 0.05, **p < 0.01, ***p < 0.001, ns meant not significant. Hypergeometric test was performed to Fig. 3F, with a significance threshold of *p* < 0.05. All experiments were performed using two biological replicates for bisulfite sequencing, and three biological replicates for RNA-seq.

### Reporting summary

Further information on research design is available in the Nature Portfolio Reporting Summary linked to this article.

## Supplementary information

Supplementary Information

Supplementary Data 1

Supplementary Data 2

Supplementary Data 3

Supplementary Data 4

Description of Additional Supplementary files

Reporting summary

## Data Availability

The sequencing data have been deposited in NCBI Sequence Read Archive (SRA) under the accession number PRJNA1146957. Source data for figures are available in Supplementary Data 1–4. Other relevant data are available from the corresponding author upon reasonable request.

## References

[1] ten Hove, C. A., Lu, K.-J. & Weijers, D. Building a plant: cell fate specification in the early *Arabidopsis* embryo. *Development* **142**, 420–430 (2015). 25605778 10.1242/dev.111500

[2] Wendrich, J. R. & Weijers, D. The Arabidopsis embryo as a miniature morphogenesis model. *N. Phytologist* **199**, 14–25 (2013). 10.1111/nph.12267 23590679

[3] Yoshida, S. et al. Genetic control of plant development by overriding a geometric division rule. *Dev. Cell* **29**, 75–87 (2014). 24684831 10.1016/j.devcel.2014.02.002

[4] Armenta-Medina, A. et al. Developmental and genomic architecture of plant embryogenesis: from model plant to crops. *Plant Commun.* **2**, 100136 (2021). 33511346 10.1016/j.xplc.2020.100136 PMC7816075

[5] Kankel, M. W. et al. Arabidopsis MET1 cytosine methyltransferase mutants. *Genetics* **163**, 1109–1122 (2003). 12663548 10.1093/genetics/163.3.1109 PMC1462485

[6] Lindroth, A. M. et al. Requirement of CHROMOMETHYLASE3 for maintenance of CpXpG methylation. *Science* **292**, 2077–2080 (2001). 11349138 10.1126/science.1059745

[7] Stroud, H. et al. Non-CG methylation patterns shape the epigenetic landscape in Arabidopsis. *Nat. Struct. Mol. Biol.* **21**, 64–72 (2014). 24336224 10.1038/nsmb.2735 PMC4103798

[8] Zemach, A. et al. The Arabidopsis nucleosome remodeler DDM1 allows DNA methyltransferases to access H1-containing heterochromatin. *Cell* **153**, 193–205 (2013). 23540698 10.1016/j.cell.2013.02.033 PMC4035305

[9] Zhang, H., Lang, Z. & Zhu, J.-K. Dynamics and function of DNA methylation in plants. *Nat. Rev. Mol. Cell Biol.* **19**, 489–506 (2018). 29784956 10.1038/s41580-018-0016-z

[10] Matzke, M. A. & Mosher, R. A. RNA-directed DNA methylation: an epigenetic pathway of increasing complexity. *Nat. Rev. Genet* **15**, 394–408 (2014). 24805120 10.1038/nrg3683

[11] Autran, D. et al. Maternal epigenetic pathways control parental contributions to Arabidopsis early embryogenesis. *Cell* **145**, 707–719 (2011). 21620136 10.1016/j.cell.2011.04.014

[12] Nodine, M. D. & Bartel, D. P. Maternal and paternal genomes contribute equally to the transcriptome of early plant embryos. *Nature* **482**, 94–97 (2012). 22266940 10.1038/nature10756 PMC3477627

[13] Zhao, P. et al. Two-step maternal-to-zygotic transition with two-phase parental genome contributions. *Dev. Cell* **49**, 882–893.e5 (2019). 31080059 10.1016/j.devcel.2019.04.016

[14] Alaniz-Fabián, J., Orozco-Nieto, A., Abreu-Goodger, C. & Gillmor, C. S. Hybridization alters maternal and paternal genome contributions to early plant embryogenesis. *Development* **149**, dev201025 (2022). 36314727 10.1242/dev.201025

[15] Xiao, W. et al. DNA methylation is critical for Arabidopsis embryogenesis and seed viability. * Plant Cell* **18**, 805–814 (2006). 16531498 10.1105/tpc.105.038836 PMC1425851

[16] Lin, J.-Y. et al. Similarity between soybean and Arabidopsis seed methylomes and loss of non-CG methylation does not affect seed development. *Proc. Natl. Acad. Sci. USA* **114**, E9730–E9739 (2017). 29078418 10.1073/pnas.1716758114 PMC5692608

[17] Pillot, M. et al. Embryo and endosperm inherit distinct chromatin and transcriptional states from the female gametes in Arabidopsis[C][W]. *Plant Cell* **22**, 307–320 (2010). 20139161 10.1105/tpc.109.071647 PMC2845419

[18] He, L. et al. DNA methylation-free Arabidopsis reveals crucial roles of DNA methylation in regulating gene expression and development. *Nat. Commun.* **13**, 1335 (2022). 35288562 10.1038/s41467-022-28940-2 PMC8921224

[19] Kawakatsu, T., Nery, J. R., Castanon, R. & Ecker, J. R. Dynamic DNA methylation reconfiguration during seed development and germination. *Genome Biol.* **18**, 171 (2017). 28911331 10.1186/s13059-017-1251-x PMC5599895

[20] Papareddy, R. K. et al. Chromatin regulates expression of small RNAs to help maintain transposon methylome homeostasis in Arabidopsis. *Genome Biol.* **21**, 251 (2020). 32943088 10.1186/s13059-020-02163-4 PMC7499886

[21] Frost, J. M., Rhee, J. H. & Choi, Y. Dynamics of DNA methylation and its impact on plant embryogenesis. *Curr. Opin. Plant Biol.* **81**, 102593 (2024). 38941722 10.1016/j.pbi.2024.102593

[22] Raissig, M. T., Gagliardini, V., Jaenisch, J., Grossniklaus, U. & Baroux, C. Efficient and rapid isolation of early-stage embryos from Arabidopsis thaliana seeds. *J. Vis. Exp.* 50371 10.3791/50371 (2013). 10.3791/50371 PMC3735039 23770918

[23] Zhou, X., Shi, C., Zhao, P. & Sun, M. Isolation of living apical and basal cell lineages of early proembryos for transcriptome analysis. *Plant Reprod.* **32**, 105–111 (2019). 30547251 10.1007/s00497-018-00353-6

[24] Liu, Q. et al. Paternal DNA methylation is remodeled to maternal levels in rice zygote. *Nat. Commun.* **14**, 6571 (2023). 37852973 10.1038/s41467-023-42394-0 PMC10584822

[25] Pignatta, D. et al. Natural epigenetic polymorphisms lead to intraspecific variation in Arabidopsis gene imprinting. *eLife* **3**, e03198 (2014). 24994762 10.7554/eLife.03198 PMC4115658

[26] Hsieh, T.-F. et al. Genome-wide demethylation of Arabidopsis endosperm. *Science* **324**, 1451–1454 (2009). 19520962 10.1126/science.1172417 PMC4044190

[27] Bouyer, D. et al. DNA methylation dynamics during early plant life. *Genome Biol.* **18**, 179 (2017). 28942733 10.1186/s13059-017-1313-0 PMC5611644

[28] Schon, M. A. & Nodine, M. D. Widespread contamination of Arabidopsis embryo and endosperm transcriptome data sets. *Plant Cell* **29**, 608–617 (2017). 28314828 10.1105/tpc.16.00845 PMC5435428

[29] Ma, X. et al. Parental variation in CHG methylation is associated with allelic-specific expression in elite hybrid rice. *Plant Physiol.* **186**, 1025–1041 (2021). 33620495 10.1093/plphys/kiab088 PMC8195538

[30] Ji, L. et al. Genome-wide reinforcement of DNA methylation occurs during somatic embryogenesis in soybean. * Plant Cell* **31**, 2315–2331 (2019). 31439802 10.1105/tpc.19.00255 PMC6790092

[31] Kumar, L. & Futschik, M. E. Mfuzz: a software package for soft clustering of microarray data. *Bioinformation* **2**, 5–7 (2007). 18084642 10.6026/97320630002005 PMC2139991

[32] Verma, S., Attuluri, V. P. S. & Robert, H. S. An essential function for auxin in embryo development. *Cold Spring Harb. Perspect. Biol.* **13**, a039966 (2021). 33431580 10.1101/cshperspect.a039966 PMC8015691

[33] Hirsch, C. D. & Springer, N. M. Transposable element influences on gene expression in plants. *Biochim. Biophys. Acta* **1860**, 157–165 (2017). 10.1016/j.bbagrm.2016.05.010 27235540

[34] Wang, X., Weigel, D. & Smith, L. M. Transposon variants and their effects on gene expression in Arabidopsis. *PLOS Genet.* **9**, e1003255 (2013). 23408902 10.1371/journal.pgen.1003255 PMC3567156

[35] Peaucelle, A. et al. Arabidopsis phyllotaxis is controlled by the methyl-esterification status of cell-wall pectins. *Curr. Biol.* **18**, 1943–1948 (2008). 19097903 10.1016/j.cub.2008.10.065

[36] Rajkumar, M. S., Gupta, K., Khemka, N. K., Garg, R. & Jain, M. DNA methylation reprogramming during seed development and its functional relevance in seed size/weight determination in chickpea. *Commun. Biol.* **3**, 1–13 (2020). 32620865 10.1038/s42003-020-1059-1 PMC7335156

[37] Makarevitch, I. et al. Transposable elements contribute to activation of maize genes in response to abiotic stress. *PLOS Genet.* **11**, e1004915 (2015). 25569788 10.1371/journal.pgen.1004915 PMC4287451

[38] Hure, V. et al. Alternative silencing states of transposable elements in Arabidopsis associated with H3K27me3. *Genome Biol.* **26**, 11 (2025). 39833858 10.1186/s13059-024-03466-6 PMC11745025

[39] Liu, W., Zhang, Y., He, H., He, G. & Deng, X. W. From hybrid genomes to heterotic trait output: challenges and opportunities. *Curr. Opin. Plant Biol.* **66**, 102193 (2022). 35219140 10.1016/j.pbi.2022.102193

[40] Klosinska, M., Picard, C. L. & Gehring, M. Conserved imprinting associated with unique epigenetic signatures in the Arabidopsis genus. *Nat. Plants* **2**, 1–8 (2016). 10.1038/nplants.2016.145 PMC5367468 27643534

[41] Raissig, M. T., Bemer, M., Baroux, C. & Grossniklaus, U. Genomic imprinting in the Arabidopsis embryo is partly regulated by PRC2. *PLOS Genet.* **9**, e1003862 (2013). 24339783 10.1371/journal.pgen.1003862 PMC3854695

[42] Huang, Y., Zhou, L., Hou, C. & Guo, D. The dynamic proteome in *Arabidopsis thaliana* early embryogenesis. *Development* **149**, dev200715 (2022). 35950926 10.1242/dev.200715

[43] Alonso-Blanco, C. et al. 1,135 genomes reveal the global pattern of polymorphism in Arabidopsis thaliana. *Cell* **166**, 481–491 (2016). 27293186 10.1016/j.cell.2016.05.063 PMC4949382

[44] Krueger, F. & Andrews, S. R. Bismark: a flexible aligner and methylation caller for Bisulfite-Seq applications. *Bioinformatics* **27**, 1571–1572 (2011). 21493656 10.1093/bioinformatics/btr167 PMC3102221

[45] Ramírez, F. et al. deepTools2: a next generation web server for deep-sequencing data analysis. *Nucleic Acids Res.* **44**, W160–W165 (2016). 27079975 10.1093/nar/gkw257 PMC4987876

[46] Akalin, A. et al. methylKit: a comprehensive R package for the analysis of genome-wide DNA methylation profiles. *Genome Biol.* **13**, R87 (2012). 23034086 10.1186/gb-2012-13-10-r87 PMC3491415

[47] Wu, T. et al. clusterProfiler 4.0: a universal enrichment tool for interpreting omics data. * Innovation* **2**, 100141 (2021). 34557778 10.1016/j.xinn.2021.100141 PMC8454663

[48] Krueger, F. & Andrews, S. R. SNPsplit: allele-specific splitting of alignments between genomes with known SNP genotypes. *F1000Res* **5**, 1479 (2016). 27429743 10.12688/f1000research.9037.1 PMC4934512

[49] Xie, W. et al. Base-resolution analyses of sequence and parent-of-origin dependent DNA methylation in the mouse genome. *Cell* **148**, 816–831 (2012). 22341451 10.1016/j.cell.2011.12.035 PMC3343639

[50] Zhang, M. et al. Genome-wide high resolution parental-specific DNA and histone methylation maps uncover patterns of imprinting regulation in maize. *Genome Res.* **24**, 167–176 (2014). 24131563 10.1101/gr.155879.113 PMC3875858

[51] Martin, M. Cutadapt removes adapter sequences from high-throughput sequencing reads. *EMBnet. J.* **17**, 10–12 (2011).

[52] Kim, D., Paggi, J. M., Park, C., Bennett, C. & Salzberg, S. L. Graph-based genome alignment and genotyping with HISAT2 and HISAT-genotype. *Nat. Biotechnol.* **37**, 907–915 (2019). 31375807 10.1038/s41587-019-0201-4 PMC7605509

[53] Liao, Y., Smyth, G. K. & Shi, W. featureCounts: an efficient general purpose program for assigning sequence reads to genomic features. *Bioinformatics* **30**, 923–930 (2014). 24227677 10.1093/bioinformatics/btt656

[54] Love, M. I., Huber, W. & Anders, S. Moderated estimation of fold change and dispersion for RNA-seq data with DESeq2. *Genome Biol.* **15**, 550 (2014). 25516281 10.1186/s13059-014-0550-8 PMC4302049

[55] Acurzio, B. et al. Zfp57 inactivation illustrates the role of ICR methylation in imprinted gene expression during neural differentiation of mouse ESCs. *Sci. Rep.* **11**, 13802 (2021). 34226608 10.1038/s41598-021-93297-3 PMC8257706

[56] Marion-Poll, L. et al. Locus specific epigenetic modalities of random allelic expression imbalance. *Nat. Commun.* **12**, 5330 (2021). 34504093 10.1038/s41467-021-25630-3 PMC8429725

[57] Shao, L. et al. Patterns of genome-wide allele-specific expression in hybrid rice and the implications on the genetic basis of heterosis. *Proc. Natl. Acad. Sci. USA* **116**, 5653–5658 (2019). 30833384 10.1073/pnas.1820513116 PMC6431163

