# Supplementary material for: DNA methylation remodeling reveals epigenetic regulation of early embryogenesis in Arabidopsis hybrid
Source: Commun Biol. 2026 May 15;9:1037. doi: 10.1038/s42003-026-10245-5 (PMC13434704; doi:10.1038/s42003-026-10245-5)
Supplement: Supplementary file 1 [file 42003_2026_10245_MOESM1_ESM.pdf]

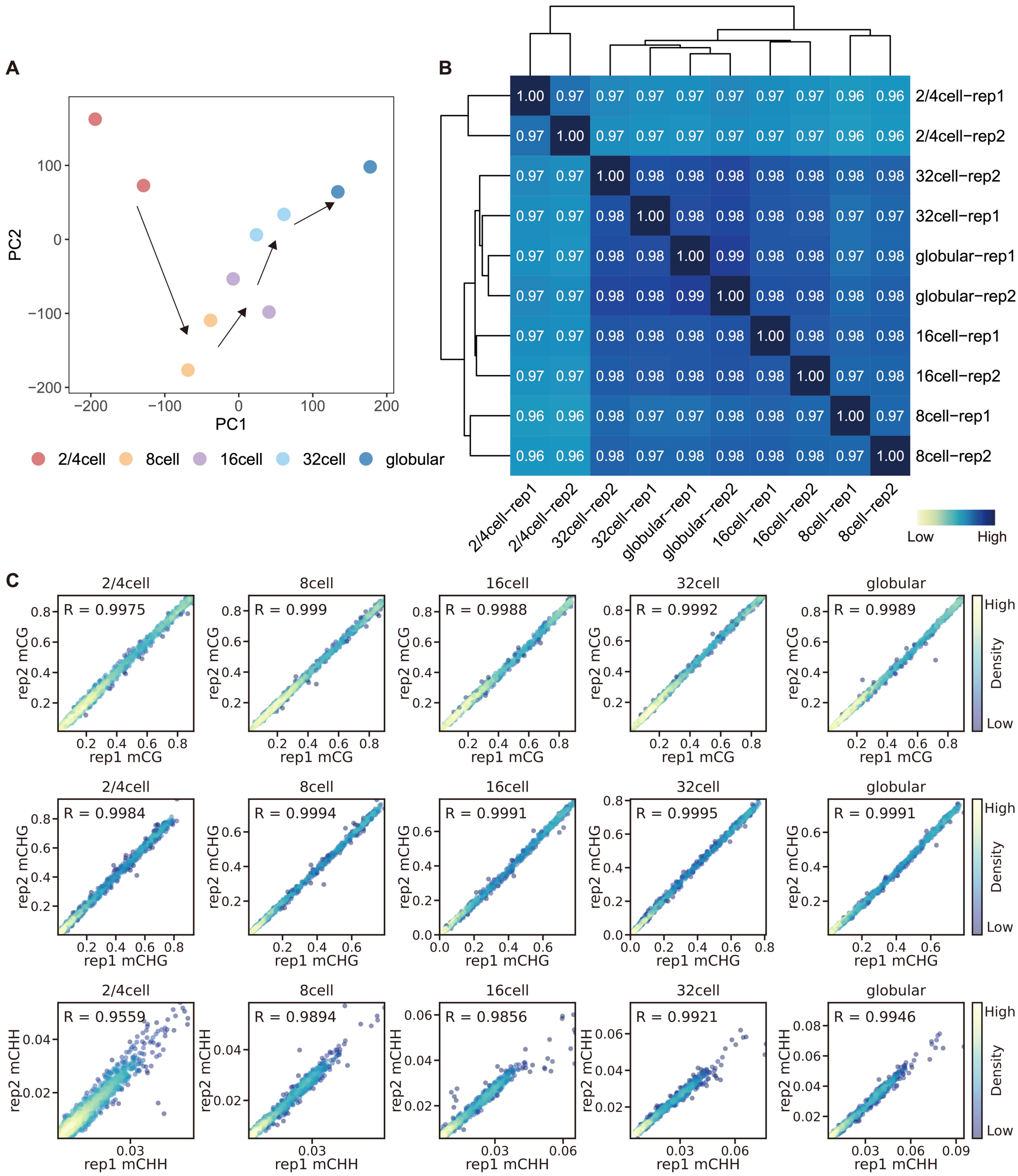

Supplementary Figure 1. Quality assessment of DNA methylation data. A PCA plot of DNA methylation levels for all cytosine contexts. Replicates from each developmental stage were displayed in the same color. Arrows represented the trajectory of development. **B** Heatmap showing the Pearson correlation coefficient of DNA methylation levels across libraries. **C** Scatterplots comparing DNA methylation levels in 50-kb bins between biological replicates for each context.

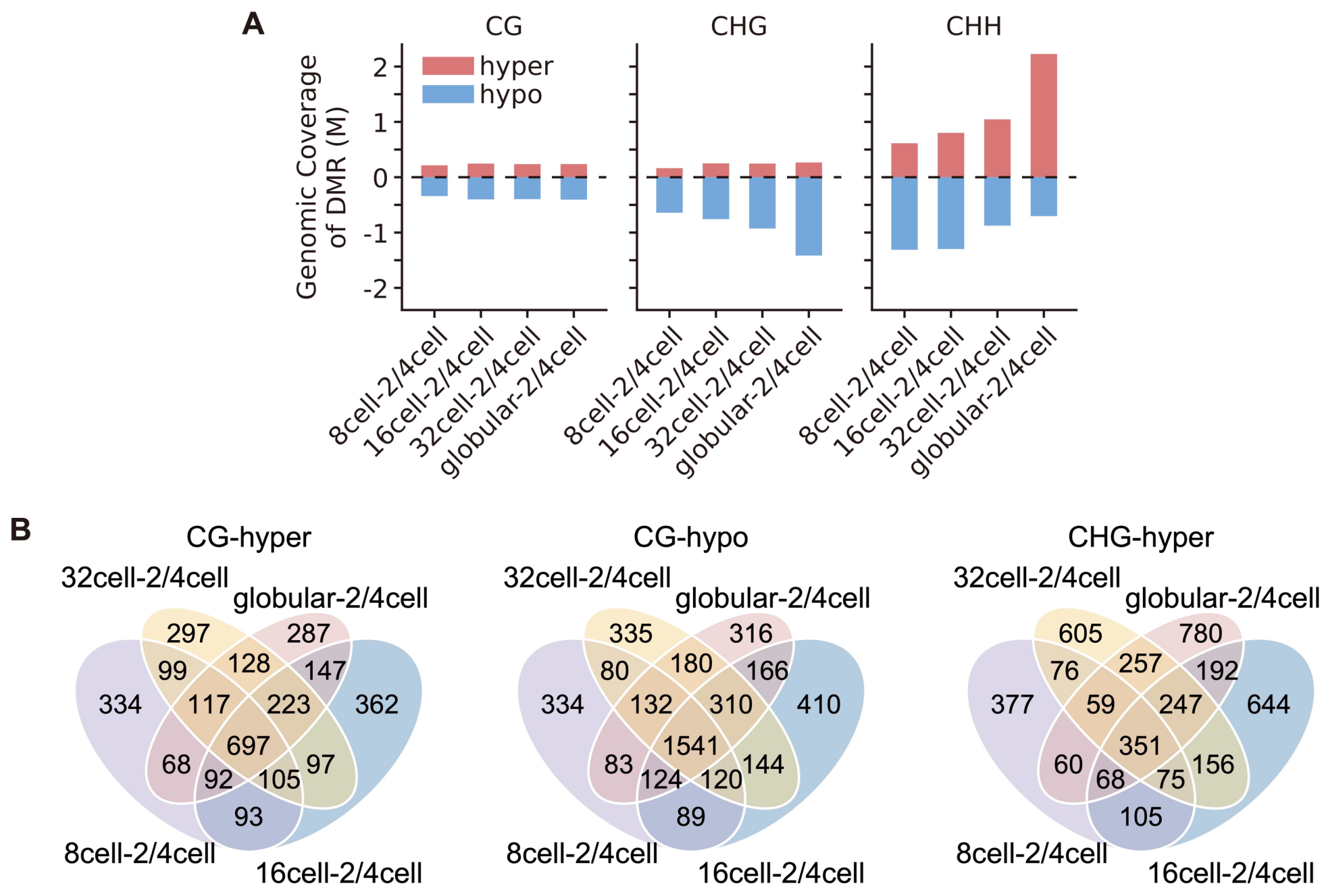

Supplementary Figure 2. DNA methylation remodeling during early embryogenesis. A Genomic coverage of identified DMRs in early embryos versus 2/4-cell stage. **B** Venn diagram showing the overlaps of identified DMRs.

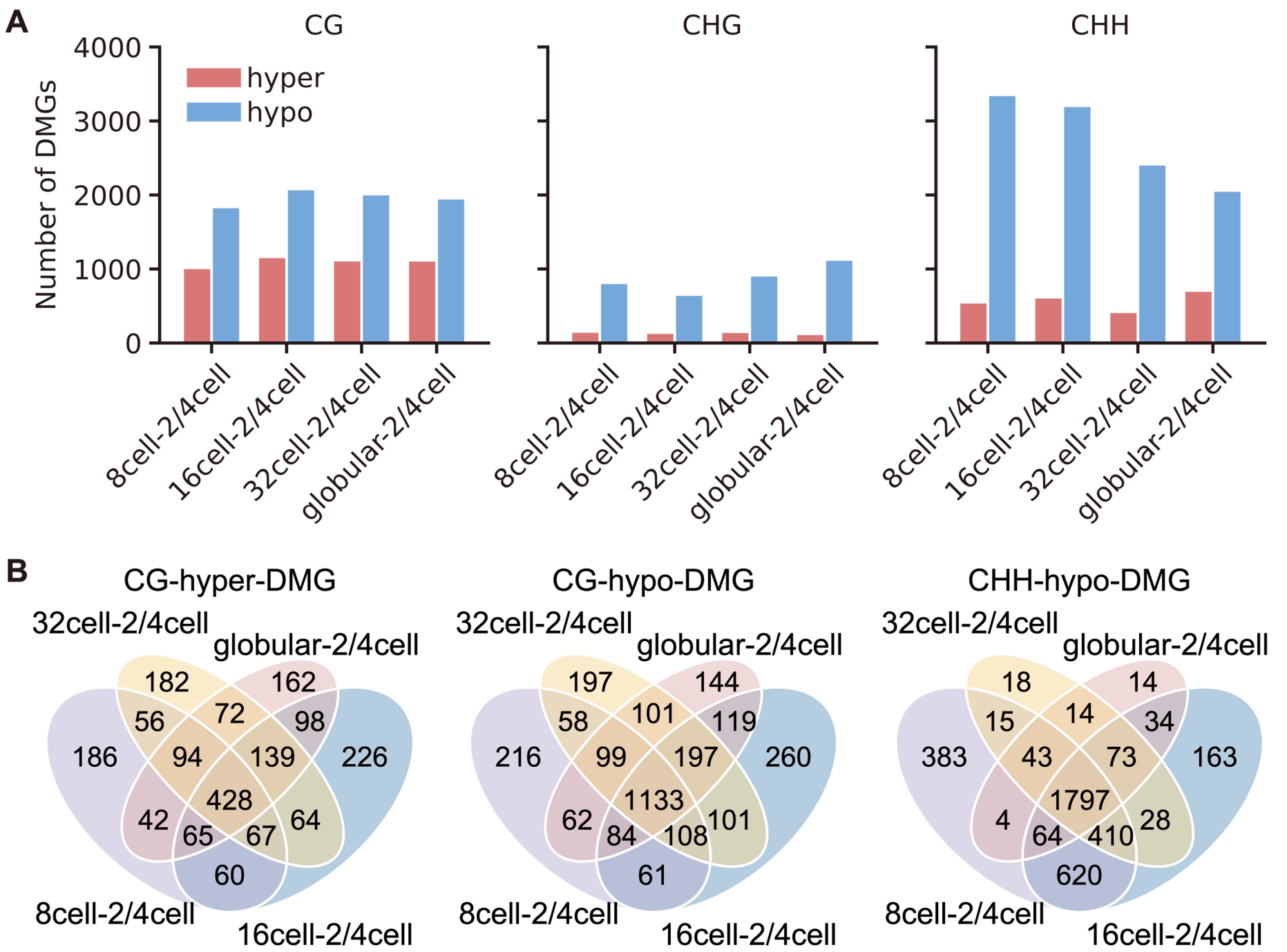

Supplementary Figure 3. Protein-coding genes relevant to differential methylation. A Number of DMR-associated genes in early embryos versus 2/4-cell stage. **B** Venn diagram showing the overlaps of identified DMGs.

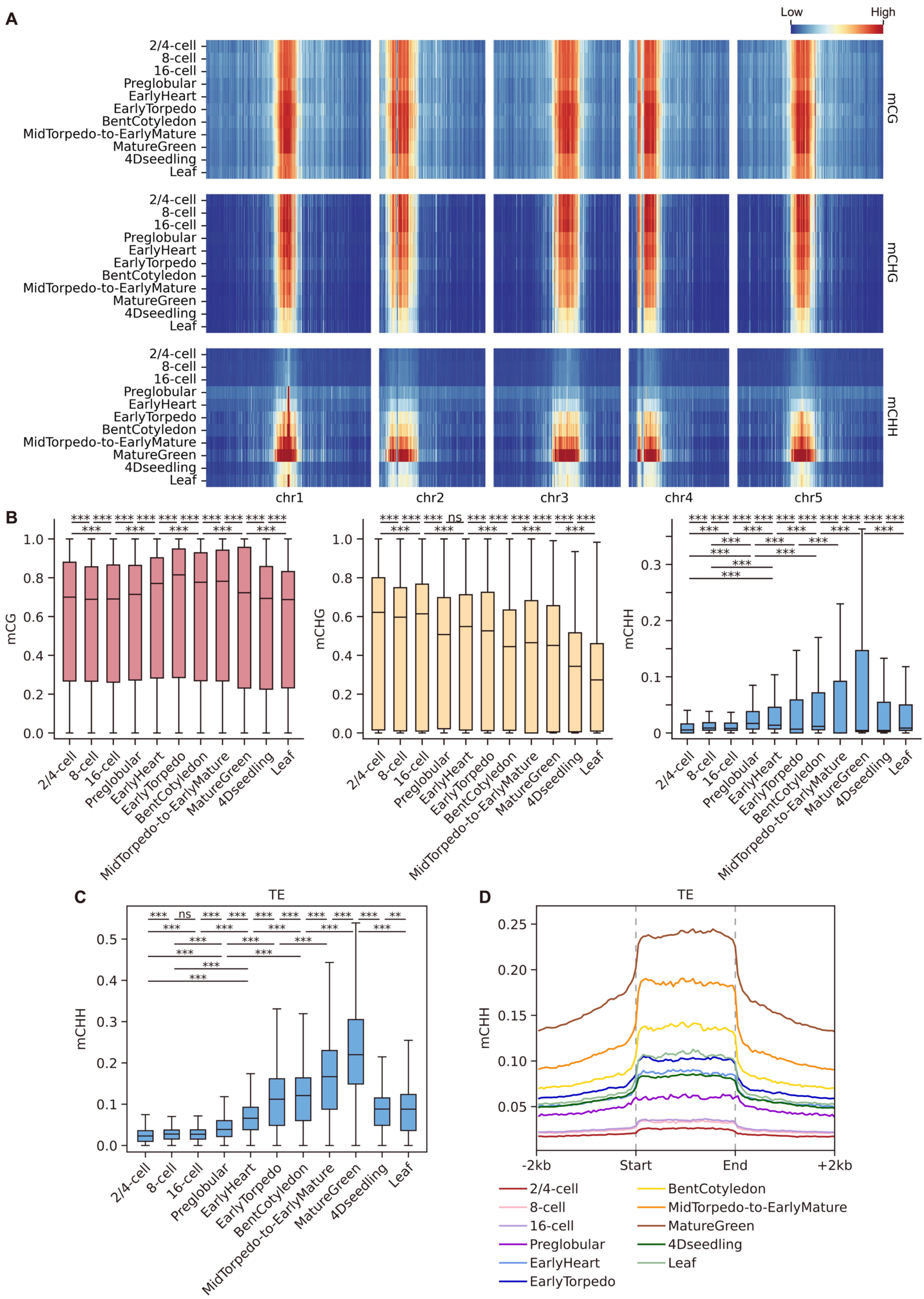

Supplementary Figure 4. Characterization of DNA methylation changes in *Arabidopsis* embryos.

A Heatmap showing genome-wide DNA methylation levels along the genome in 100-kb bins. **B** Boxplots showing DNA methylation levels in 100-bp bins. **C** Boxplots showing CHH methylation levels for TEs. Significance was assessed by Mann-Whitney U test; thresholds: *p<0.05, **p<0.01, ***p<0.001, ns meant not significant. **D** Metaplots profiling CHH methylation levels across TEs. Publicly available methylomes for embryos at pre-globular[1], early heart[1], early torpedo[2], bent cotyledon[1], mid-torpedo to early mature[3], and mature green[4] stages, as well as seedling[4] and leaf[1], were downloaded and compared with our methylation data from early embryos at 2/4-cell, 8-cell and 16-cell stages.

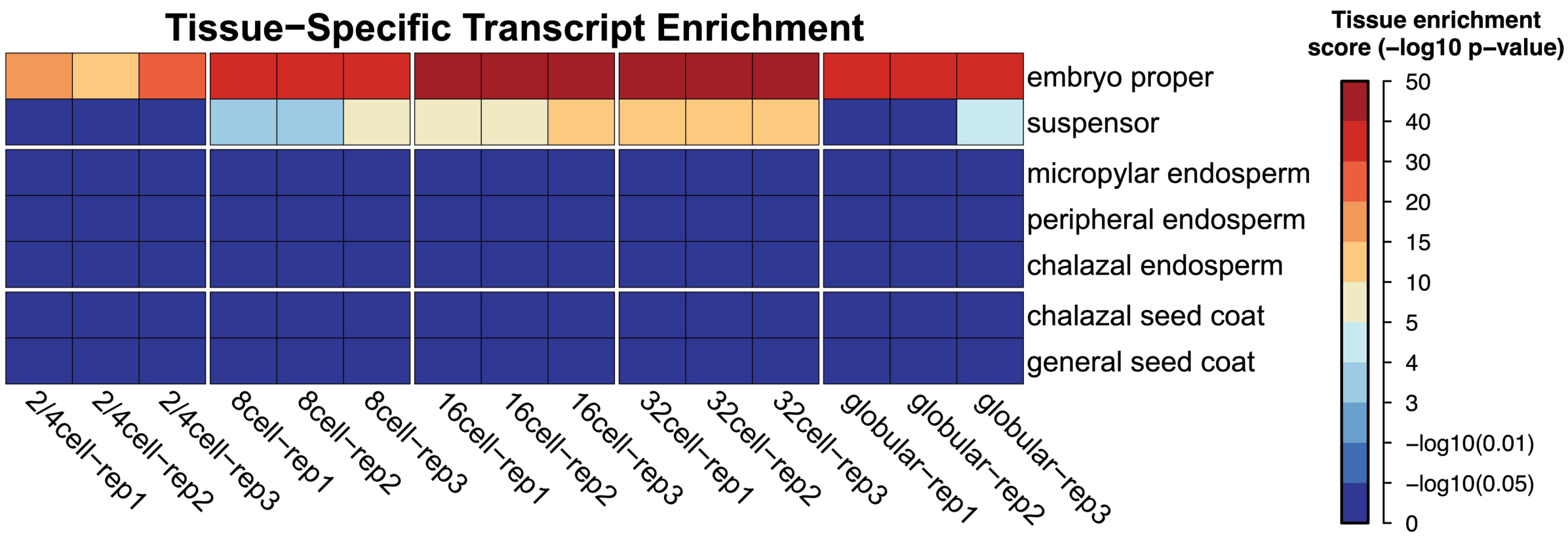

Supplementary Figure 5. Estimation of RNA contamination in embryonic transcriptome data using tissue enrichment test.

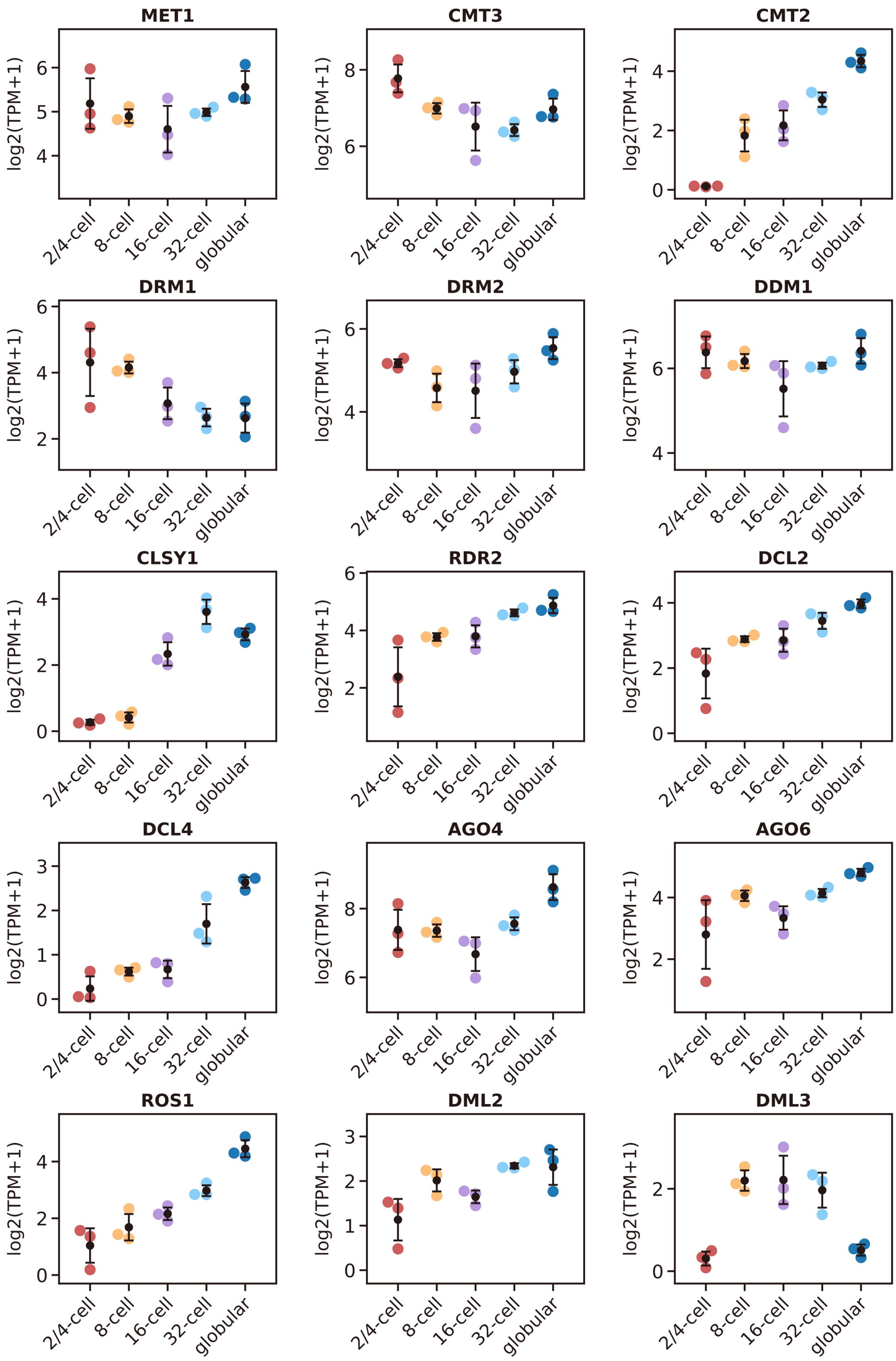

Supplementary Figure 6. Expression of genes participating in the establishment, maintenance and removal of DNA Methylation. Black error bars indicated the mean ± standard deviation (n=3).

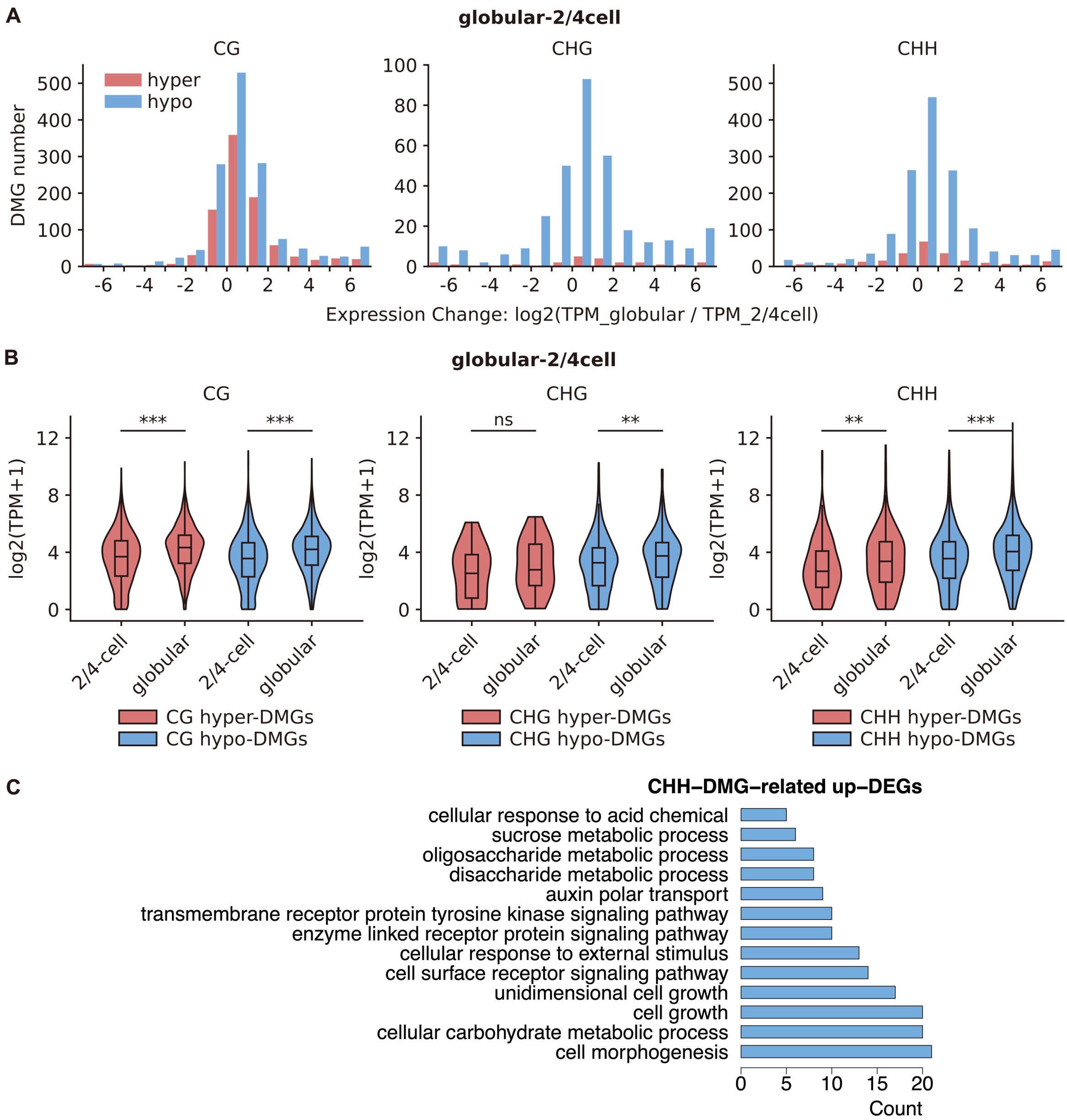

Supplementary Figure 7. Relationship between differential methylation and gene expression. A Number of hyper- and hypo-DMGs identified in globular versus 2/4-cell stage, whose expression changes were distributed in distinct intervals, according to the log_2FoldChange. **B** Boxplots showing the expression levels of genes with DMRs identified in globular versus 2/4-cell stage. Significance was assessed by Mann-Whitney U test; thresholds: $*p<0.05$, $**p<0.01$, $***p<0.001$, ns meant not significant. **C** GO enrichment analysis of up-regulated DEGs that were overlapped with CHH DMRs (FDR<0.05). The number of genes associated with each GO term was shown in the x axis with blue.

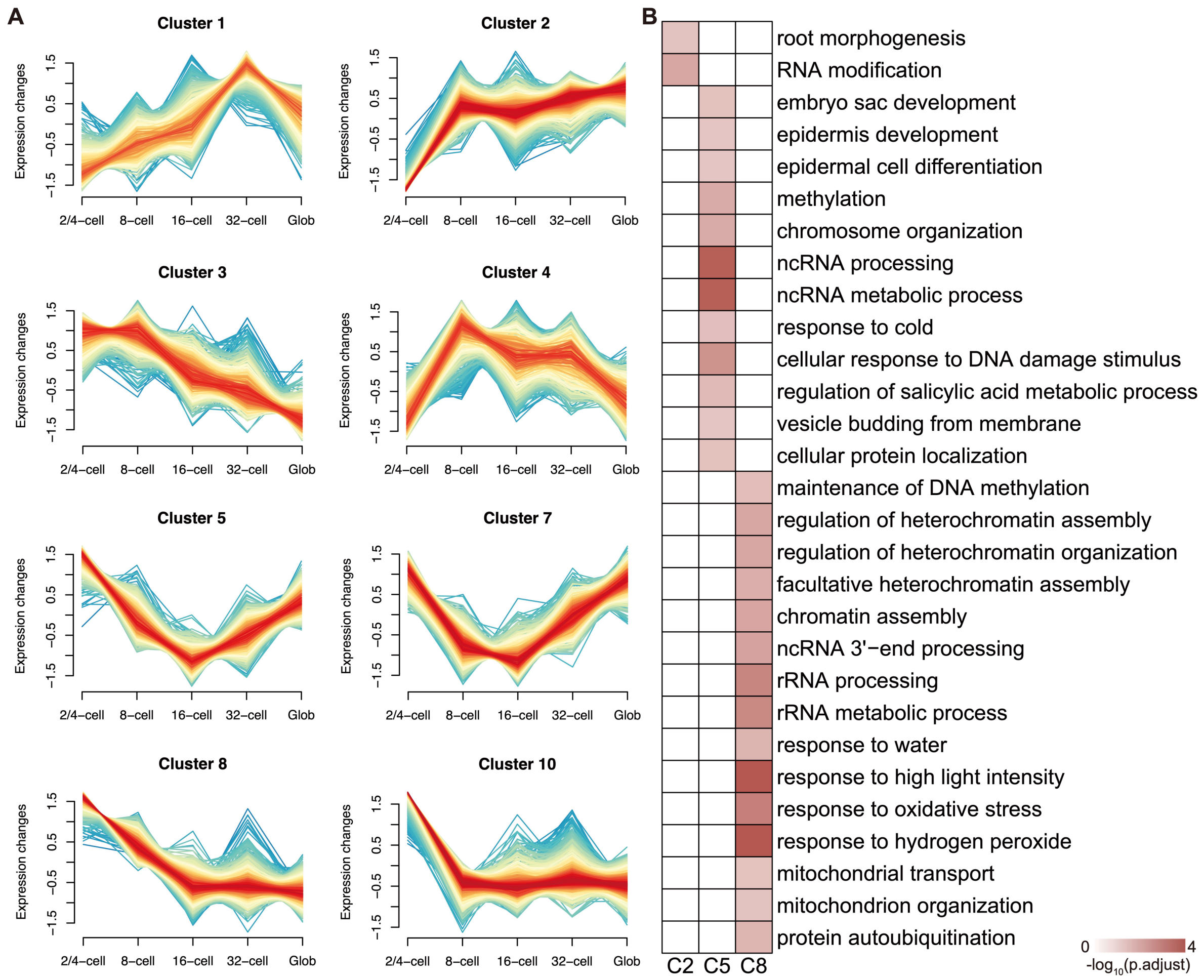

Supplementary Figure 8. The dynamic embryonic transcriptome in *Arabidopsis* early embryos. A Time-series expression changes of DEGs in the remaining 8 clusters. **B** GO enrichment analysis of DEGs (FDR<0.05). The colorbar represented $-\log_{10}$(FDR).

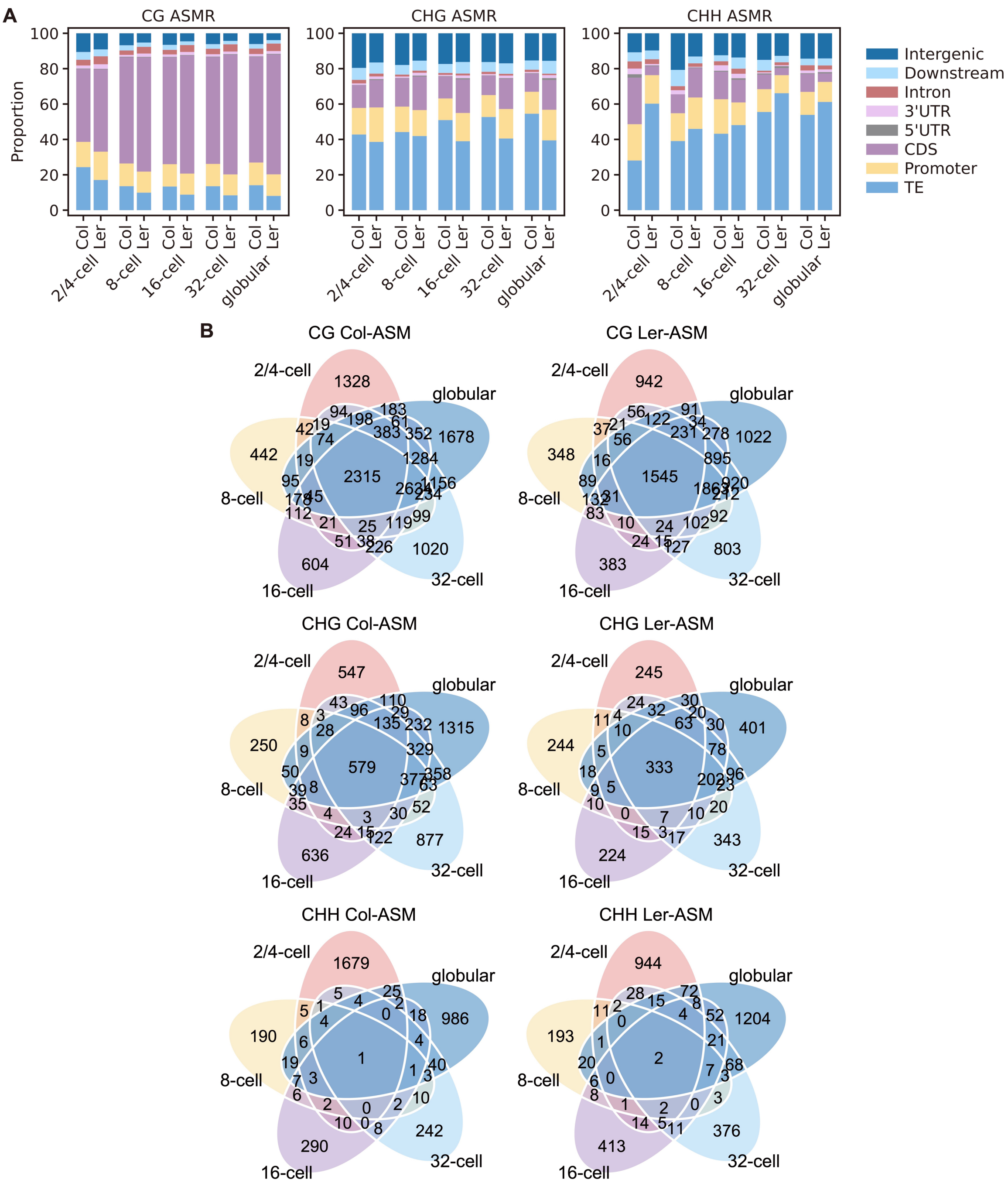

Supplementary Figure 9. Dynamics of ASMRs during early embryogenesis. A Annotation of ASMRs distribution with genomic features. **B** Venn diagram showing the overlaps of identified ASMRs.

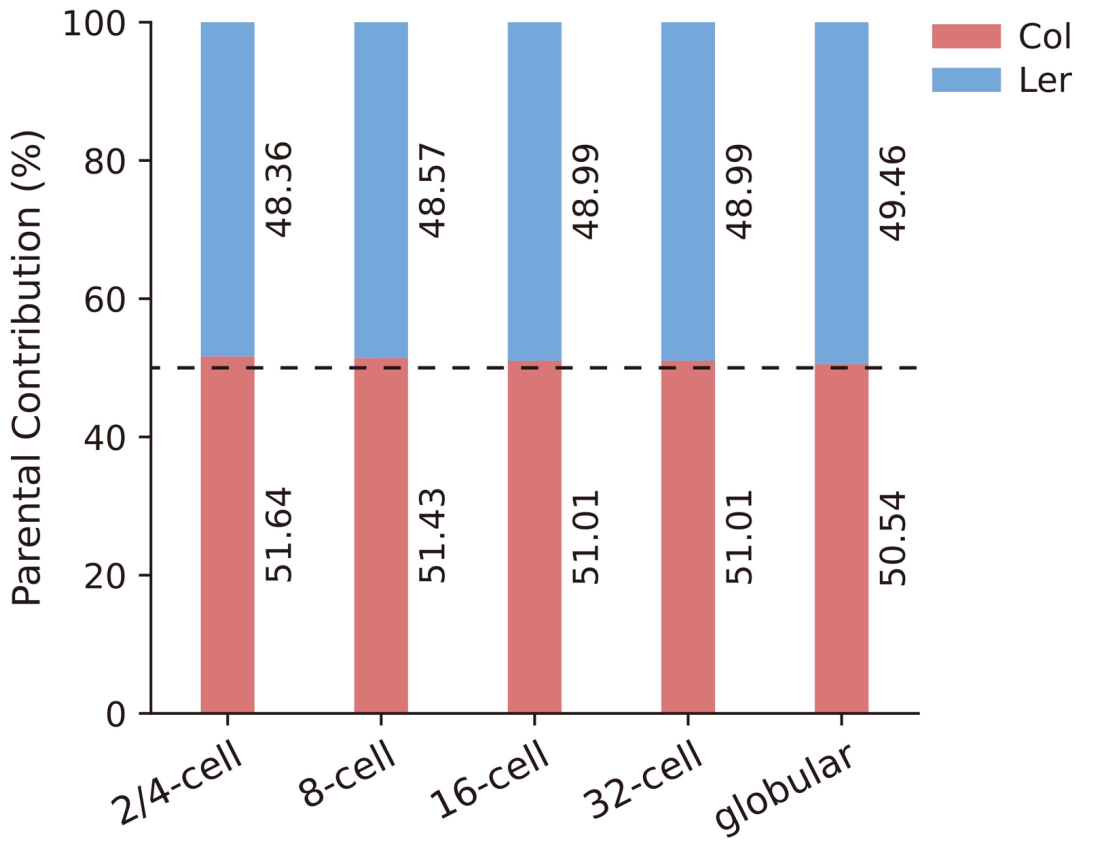

Supplementary Figure 10. Parental genome contributions to early embryonic transcriptomes.

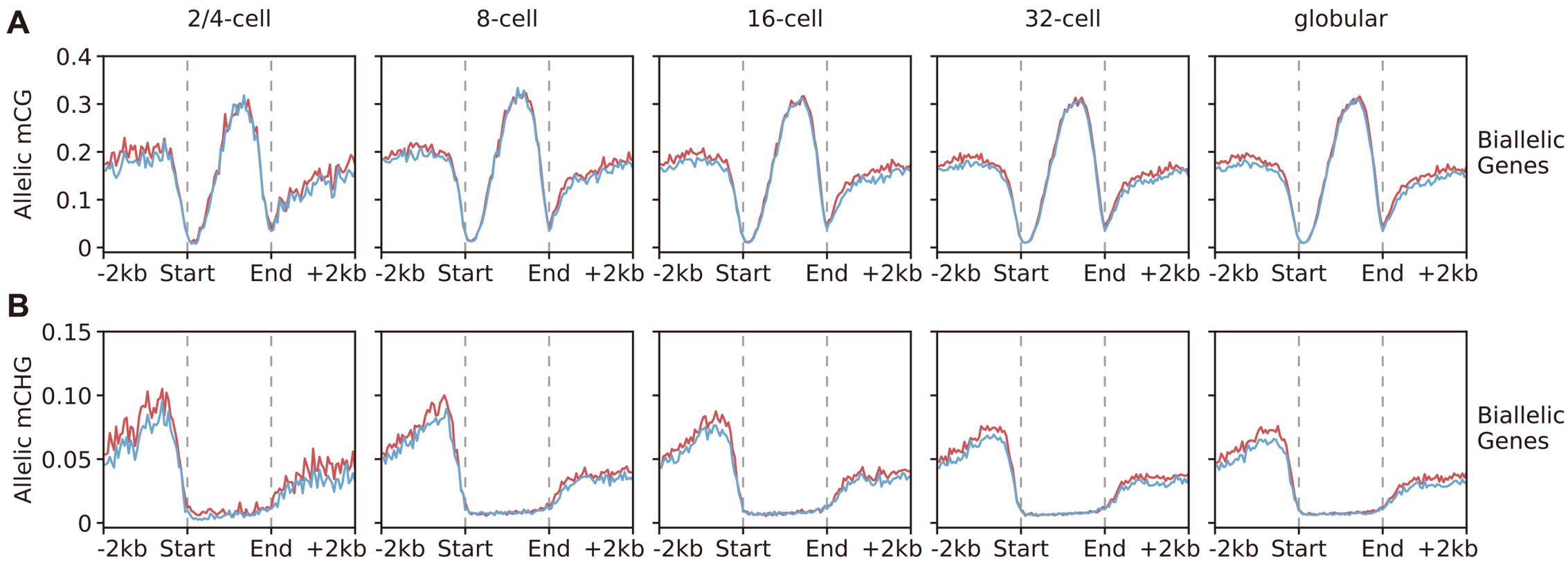

Supplementary Figure 11. Allelic DNA methylation for biallelic genes in early embryos. A-B Metaplots showing allelic CG (**A**) and CHG (**B**) methylation levels across biallelic expressed genes identified in early embryos, respectively.

Supplementary Table 1. Embryo isolation for library preparation.

stages	number of embryos used for BS-seq	number of embryos used for RNA-seq	hours after pollination (HAP)
2/4-cell	50, 50	40, 40, 40	~48 HAP
8-cell	48, 30	40, 40, 40	~60 HAP
16-cell	35, 35	30, 30, 30	~70 HAP
32-cell	15, 19	30, 30, 30	~72 HAP
Globular	20, 20	20, 20, 20	~84 HAP

Supplementary References

1. Papareddy, R. K. *et al.* Chromatin regulates expression of small RNAs to help maintain transposon methylome homeostasis in Arabidopsis. *Genome Biol* **21**, 251 (2020).
2. Pignatta, D. *et al.* Natural epigenetic polymorphisms lead to intraspecific variation in Arabidopsis gene imprinting. *eLife* **3**, e03198 (2014).
3. Hsieh, T.-F. *et al.* Genome-Wide Demethylation of Arabidopsis Endosperm. *Science* **324**, 1451–1454 (2009).
4. Bouyer, D. *et al.* DNA methylation dynamics during early plant life. *Genome Biology* **18**, 179 (2017).
