# Supplementary material for: DNA methylation remodeling reveals epigenetic regulation of early embryogenesis in Arabidopsis hybrid
Source: Commun Biol. 2026 May 15;9:1037. doi: 10.1038/s42003-026-10245-5 (PMC13434704; doi:10.1038/s42003-026-10245-5)
Supplement: Supplementary file 3 [file 42003_2026_10245_MOESM6_ESM.docx]

**Description of Additional Supplementary Files**

File name: Supplementary Data 1

Description: Differentially methylated regions (DMRs) identified in early embryos.

File name: Supplementary Data 2

Description: List of differentially expressed genes (DEGs) in 10 clusters.

File name: Supplementary Data 3

Description: Identification of regions with allele specific methylation (ASMRs) in early embryos.

File name: Supplementary Data 4

Description: Identification of genes with allele specific expression (ASEGs) in early embryos.
